# Supplementary material for: Ketogenic diet suppresses colorectal cancer through the gut microbiome long chain fatty acid stearate
Source: Nat Commun. 2025 Feb 20;16:1792. doi: 10.1038/s41467-025-56678-0 (PMC11842570; doi:10.1038/s41467-025-56678-0)
Supplement: Supplementary file 1 — Supplementary Information File [file 41467_2025_56678_MOESM1_ESM.pdf]

Supplementary Information File

Ketogenic diet suppresses colorectal cancer through the gut microbiome long chain fatty acid stearate

Mina Tsenkova[1], Madita Brauer[1,2], Vitaly I. Pozdeev[1], Marat Kasakin[3], Susheel Bhanu Busi[3,4], Maryse Schmoetten[1], Dean Cheung[1], Marianne Meyers[1], Fabien Rodriguez[1], Anthoula Gaigneaux[1], Eric Koncina[1], Cedric Gilson[1], Lisa Schlicker[3], Diran Herebian[5], Martine Schmitz[1], Laura de Nies[3], Ertan Mayatepek[5], Serge Haan[1], Carine de Beaufort[6], Thorsten Cramer[7], Johannes Meiser[8], Carole L. Linster[3], Paul Wilmes[1,3], Elisabeth Letellier[1,*]

[1] Department of Life Sciences and Medicine, Faculty of Science, Technology and Medicine, University of Luxembourg, Esch-sur-Alzette, Luxembourg

[2] Institute for Advanced Studies, University of Luxembourg, Esch-sur-Alzette, Luxembourg

[3] Centre for Systems Biomedicine, University of Luxembourg, Esch-sur-Alzette, Luxembourg

[4] UK Centre for Ecology and Hydrology, Wallingford, United Kingdom

[5] Department of General Pediatrics, Neonatology and Pediatric Cardiology, Medical Faculty and University Hospital Düsseldorf, Heinrich Heine University, Düsseldorf, Germany

[6] Pediatric Clinic, Centre Hospitalier de Luxembourg, Luxembourg, Luxembourg

[7] Department of General, Visceral, Children and Transplantation Surgery, RWTH University Hospital Aachen, Aachen, Germany

[8] Department of Cancer Research (DOCR), Luxembourg Institute of Health, Luxembourg, Luxembourg

[*]Corresponding author: elisabeth.letellier@uni.lu

a

Donor number	1	2	3	4	5
Sex	F	F	M	F	M
Age (years)	60	62	43	58	57

b

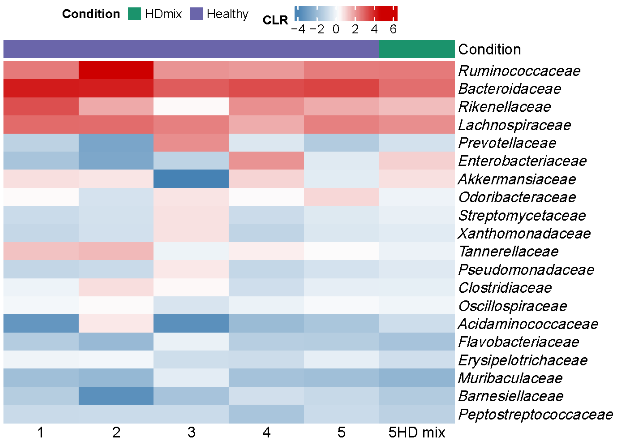

c

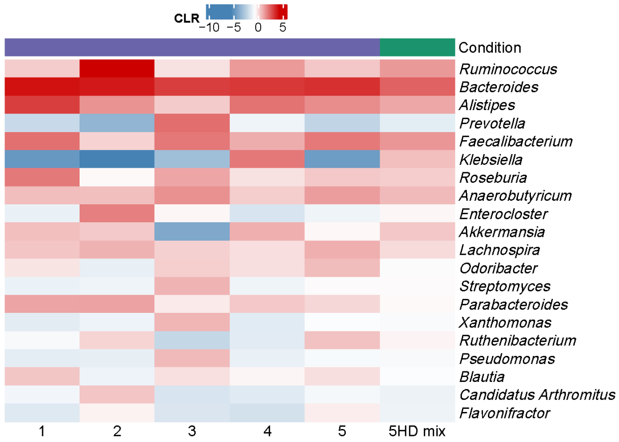

d

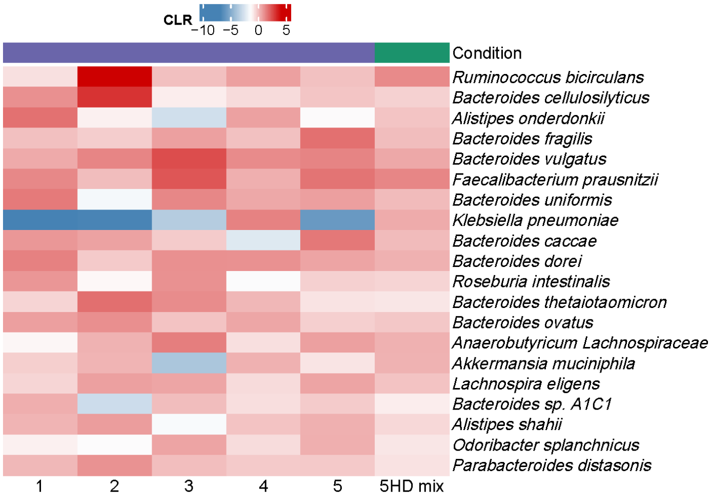

e

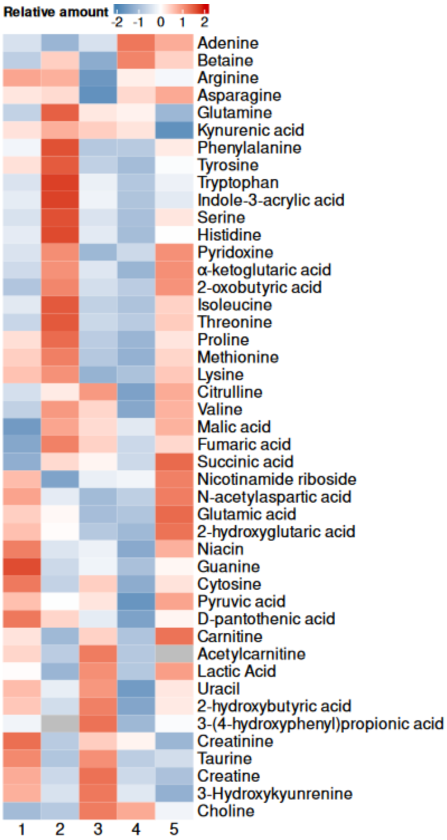

Supplementary figure 1. Characterization of donor fecal samples. | a. Metadata of fecal sample donors. M=male, F=female. **b-d.** Relative abundance of the top 20 most abundant bacterial families (**b**), genera (**c**) and species (**d**) in human stool samples (CLR units). **e.** Relative metabolite levels detected in human fecal samples, analyzed by untargeted LC-MS. Source data are provided as a Source Data file.

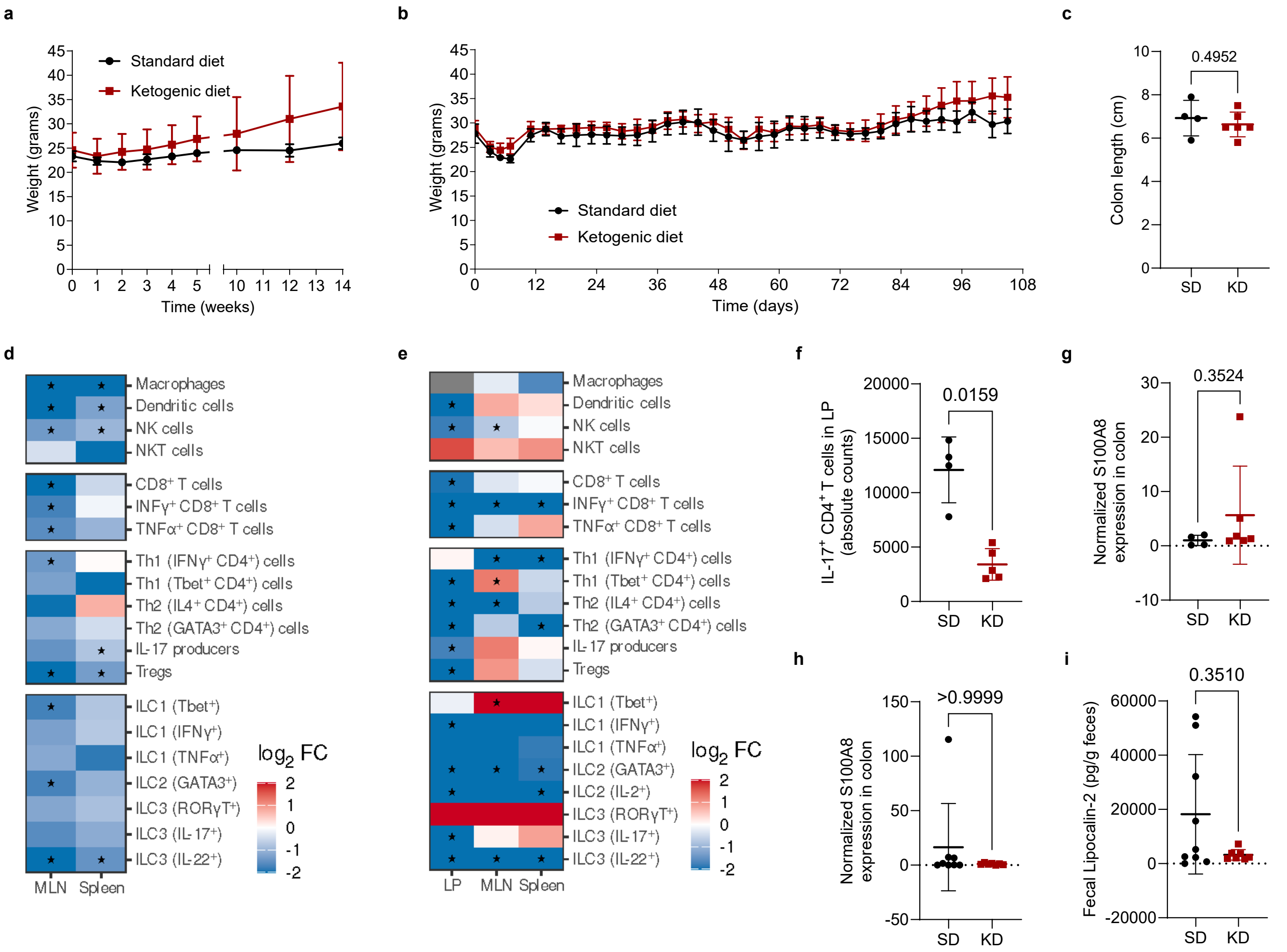

Supplementary Figure 2. Disease characteristics in an inflammatory mouse model of CRC| a-b. Mouse body weight (grams) over time (weeks) in the GF (**a**) and SPF (**b**) dietary experiments. Weight was not recorded between week five and ten (**a**) as no scale was available in the facility. **c.** Colon length (cm) in the GF dietary experiment at experimental endpoint. **d-e.** Heatmap of KD-induced changes in the immune cell compartment in relation to SD (log2foldchange) in the GF (**d**) and SPF (**e**) diet experiments as detected by flow cytometry. Stars overlaid on the heatmaps indicate *p<0.05, least squared means analysis. **f.** CD4+ IL-17+ T cells (counts) in the colons of SD and KD mice. **g-h**. Expression of S1008A in the colons of SD and KD mice in the GF (**g**) and SPF (**h**) dietary experiments, as detected by qPCR. **i**. Fecal lipocalin-2 levels (pg per gram of feces) in the SPF dietary experiment as detected by ELISA. Data is shown as mean±SD in (**a**), (**b**), (**c**), (**f**), (**g**), (**h**), and (**i**), Mann-Whitney U test. Data in (**a**), (**c**), (**d**), (**e**), (**f**) and (**g**) shows n=4 mice in the SD-fed condition and n=5 or 6 mice in the KD-fed condition from one experiment in the GF facility. Data in (**b**), (**h**) and (**i**) shows n=7 or 8 and 9 mice in the KD-fed condition and the SD-fed condition respectively, pooled from two independent experiments in the SPF facility. Source data are provided as a Source Data file.

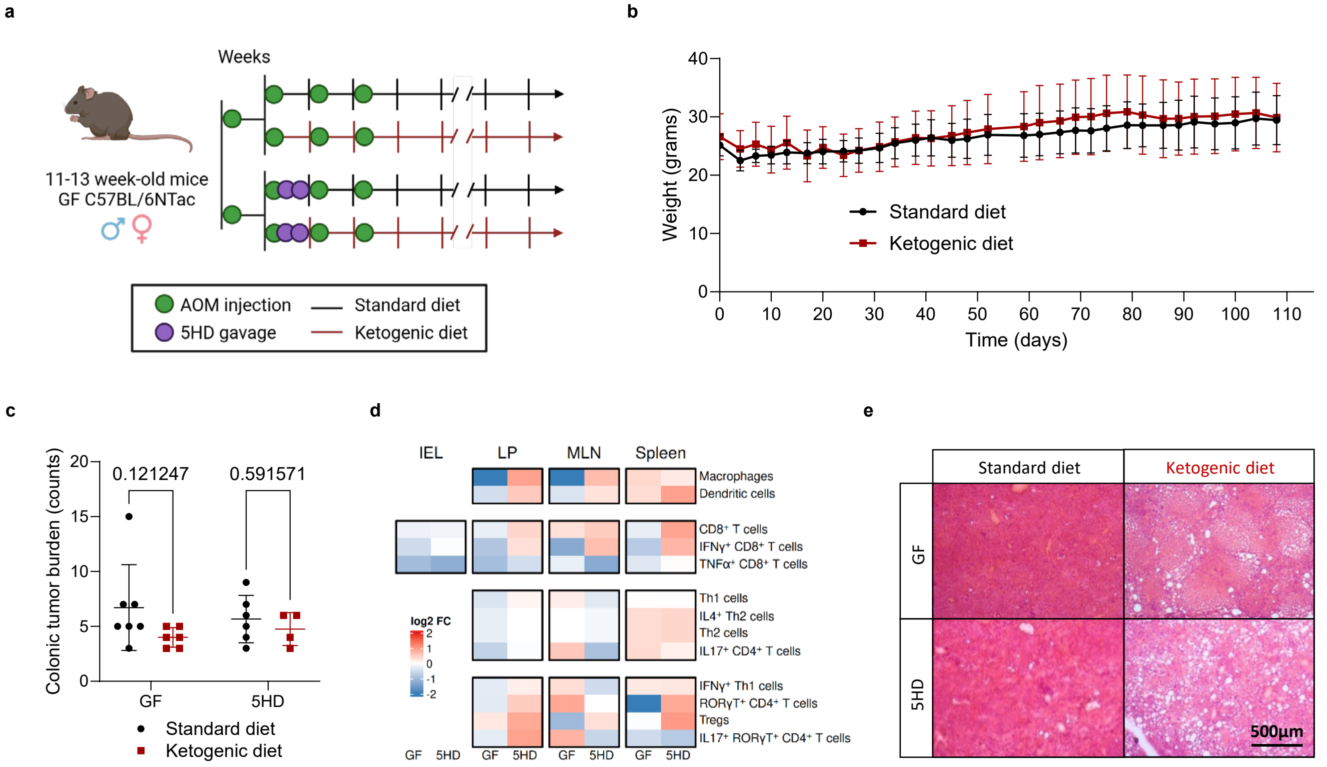

Supplementary Figure 3. Characterization of a low-grade inflammatory mouse model of CRC | a. Schematic representation of the dietary AOM experimental setup in the GF facility (Created in BioRender. Rodriguez, F. (2024) BioRender.com/w69g496). **b.** Mouse body weight (grams) over time (days) in mice administered 5HD. **c.** Colonic tumor burden at endpoint. **d.** Heatmap of immune cell population phenotyping of intra-epithelial lymphocytes (IEL), lamina propria (LP), mesenteric lymph nodes (MLN) and spleens of KD-fed mice in relation to SD-fed mice in GF mice (GF) or in mice gavaged with a mixture of stool samples from five healthy donors (5HD). Least-squared means analysis was performed on each combination of tissue and cell immune cell type using a linear model, however no statistically significant differences were found. **g.** One representative image of a section of liver stained with H&E per condition. Three sections were stained per mouse (n=7 and 6 mice in the GF SD and KD conditions respectively and n=6 and 4 mice in the 5HD SD and KD conditions, respectively) with similar results. Scale bar=500µm. Data in (**b**) and (**c**) shown as mean±SD. Mann-Whitney U test in (**c**)**.** Data in (**b**), (**c**) and (**d**) shows n=7 and 6 mice in the GF SD and KD conditions respectively and n=6 and 4 mice in the 5HD SD and KD conditions, respectively. Source data are provided as a Source Data file.

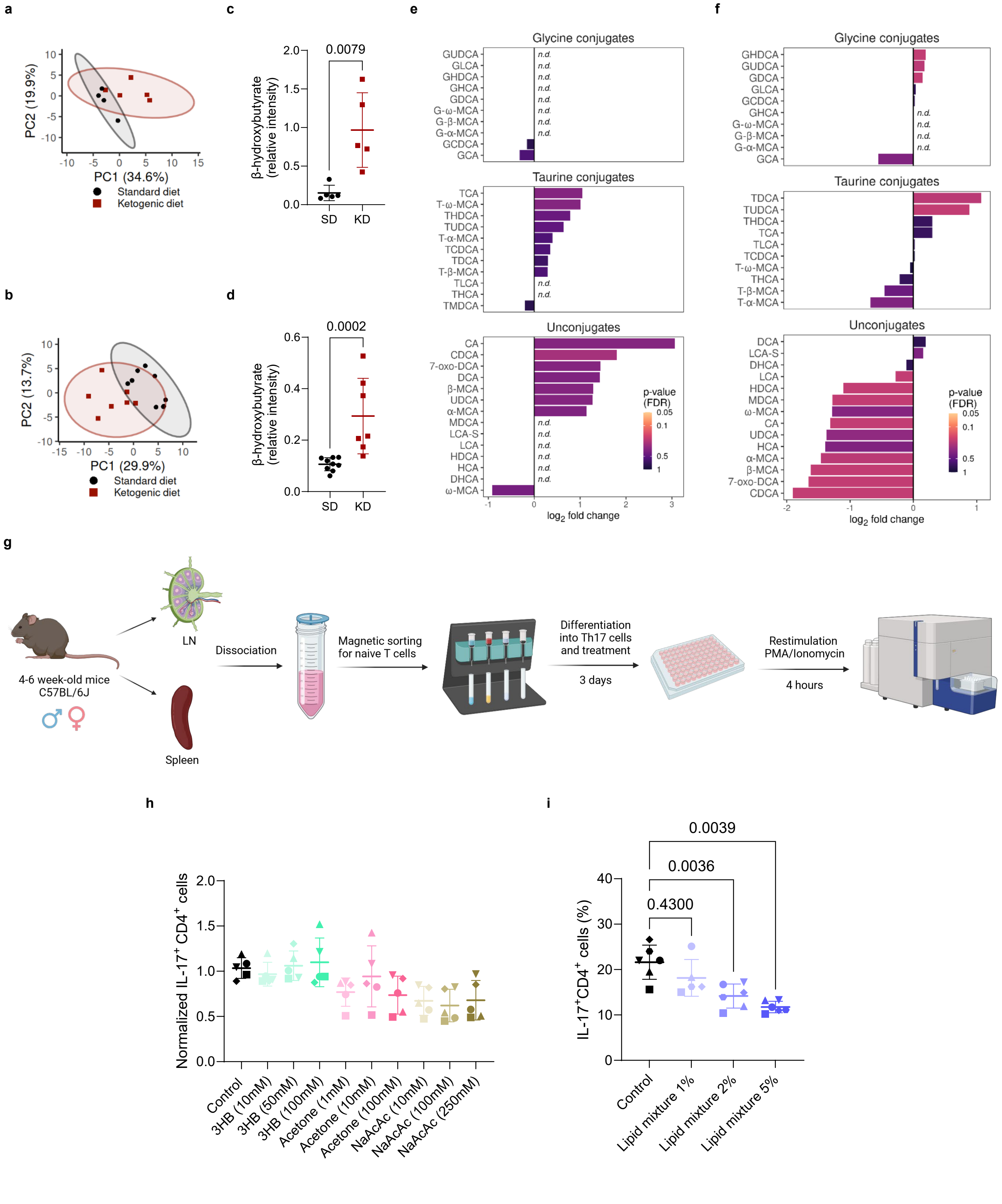

Supplementary Figure 4. Characterization of circulating metabolites during KD feeding and their importance in CRC | a-b. PCA (% confidence interval = 95) of plasma metabolites from mice in the GF (**a**) and SPF (**b**) diet experiments, as analyzed by GC-MS (**a**) and united GC-MS and LC-MS analyses (**b**). **c-d.** Relative intensity of β-HB in plasma from mice in the GF (**c**) and SPF (**d**) diet experiments, as analyzed by GC-MS. **e-f.** Log_2foldchange of identified plasma bile acids in KD-fed mice in relation to SD-fed mice in the GF (**e**) and SPF (**f**) facilities, as analyzed UHPLC-MS. **g.** Schematic representation of the experimental setup of a Th17 cell differentiation assay (Created in BioRender. Rodriguez, F. (2024) BioRender.com/w69g496). **h-i.** Th17 differentiation upon treatment with β-HB (10mM, 50mM, 100mM), acetone (1mM, 10mM, 100mM) or sodium acetoacetate (10mM, 100mM, 250mM) in (**h**), a lipid mixture (1%, 2%, 5%) in (**i**). Data in (**c**), (**d**), (**h**) and (**i**) is shown as mean±SD. Two-tailed Mann-Whitney U test in (**c**) and (**d**); Two-tailed unpaired T test with FDR correction in (**e**) and (**f**); Paired ordinary one-way ANOVA in (**h**) and (**i**). Paired ordinary one-way ANOVA comparing each set of ketone body dilutions to the control yielded no statistical significance in (**h**). Data in (**a**), (**c**) and (**e**) shows n=5 mice per condition. Data in (**b**), (**d**) and (**e**) shows n=9 mice in the SD-fed condition and n=7 mice in the KD-fed condition. Data in (**h**) shows n=5 biological replicates (mice) indicated by different datapoint shapes, from one experiment. Data in (**i**) shows n=6 biological replicates (mice) indicated by different datapoint shapes, pooled from two experiments. Abbreviations: not detected (n.d.), cholic acid (CA), hyocholic acid (HCA), chenodeoxycholic acid (CDCA), deoxycholic acid (DCA), 7-oxo-deoxycholic acid (7-oxo-DCA), murideoxycholic acid (MDCA), lithocholic acid (LCA), ursodeoxycholic acid (UDCA), hyodeoxycholic acid (HDCA), muricholic acids (α-MCA, β-MCA and ω-MCA), taurocholic acid (TCA), taurohyocholic acid (THCA), taurochenodeoxycholic acid (TCDCA), taurodeoxycholic acid (TDCA), taurolithocholic acid (TLCA), tauroursodeoxycholic acid (TUDCA), taurohyodeoxycholic acid (THDCA), tauro-α-muricholic acid (T-α-MCA), tauro-β-muricholic acid (T-β-MCA) and tauro-ω-muricholic acid (T-ω-MCA). Source data are provided as a Source Data file.

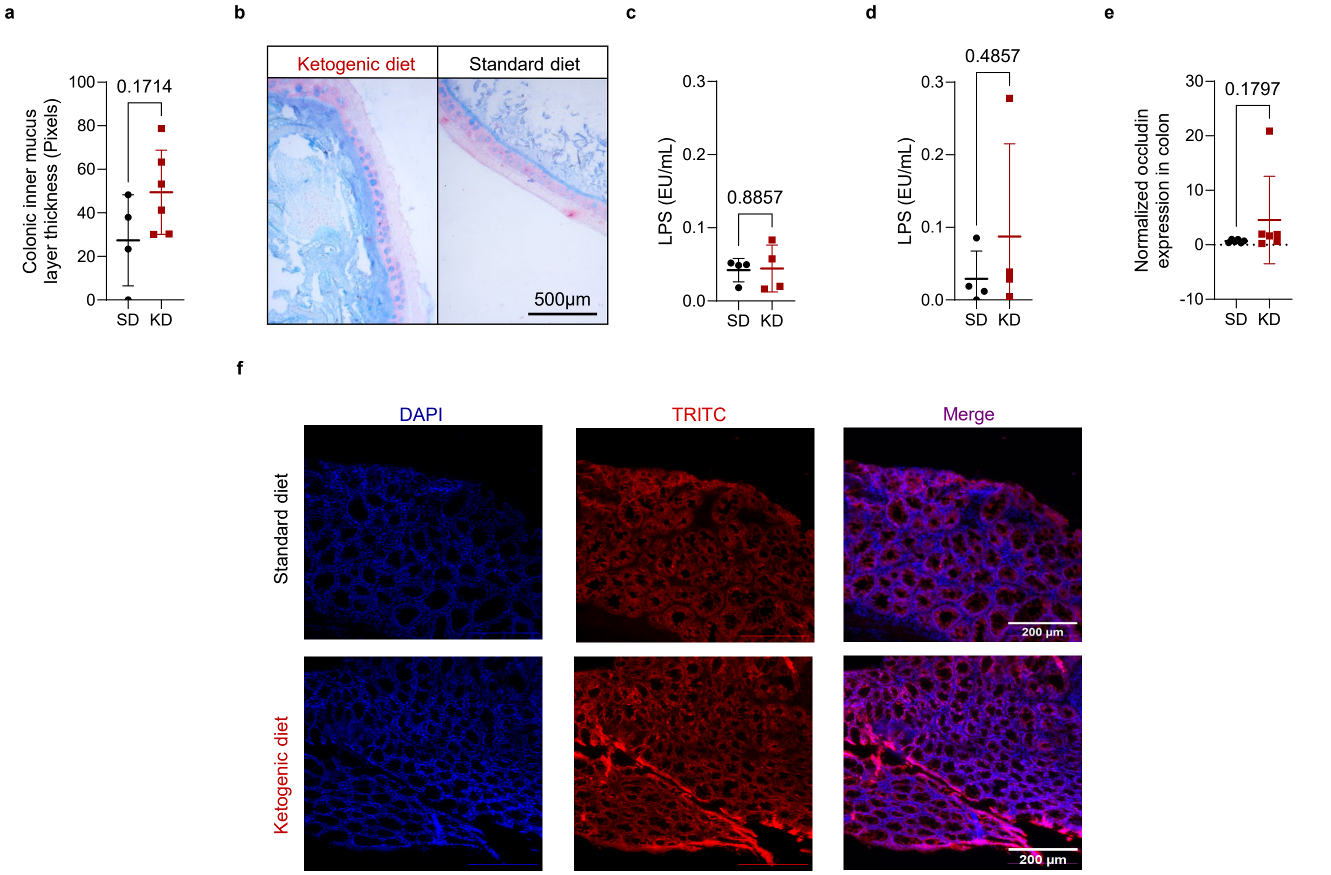

Supplementary Figure 5. | a-b. Colonic inner mucus layer thickness (in pixels, **a**) and representative images of Alcian Blue staining of colonic cryosections (**b**) in GF KD-fed and SD-fed mice. **c-d.** LPS (endotoxin units (EU) per milliliter) in plasma from GF (**c**) and SPF (**d**) KD-fed mice and SD-fed mice measured by ELISA. **e-f.** Colonic occludin gene expression (**e**) and protein expression (**f**) in KD and SD mice in the SPF dietary experiment, as measured by qPCR (**e**) and immunofluorescent staining (occludin-TRITC in red and DAPI nuclear stain in blue, one representative colon per condition is shown (two sections were stained per mouse (n=5 SD and n=4 KD mice) with similar results), scale bar = 200 µM, **f**). Two-tailed Mann-Whitney U test in (**a**), (**c**), (**d**), (**e**) and (**g**). Data in (**a**) shows n=4 SD and n=6 KD mice. Data in (**c**) and (**d**) shows n=4 mice per condition. Data in (**e**) shows n=6 mice per condition. Source data are provided as a Source Data file.

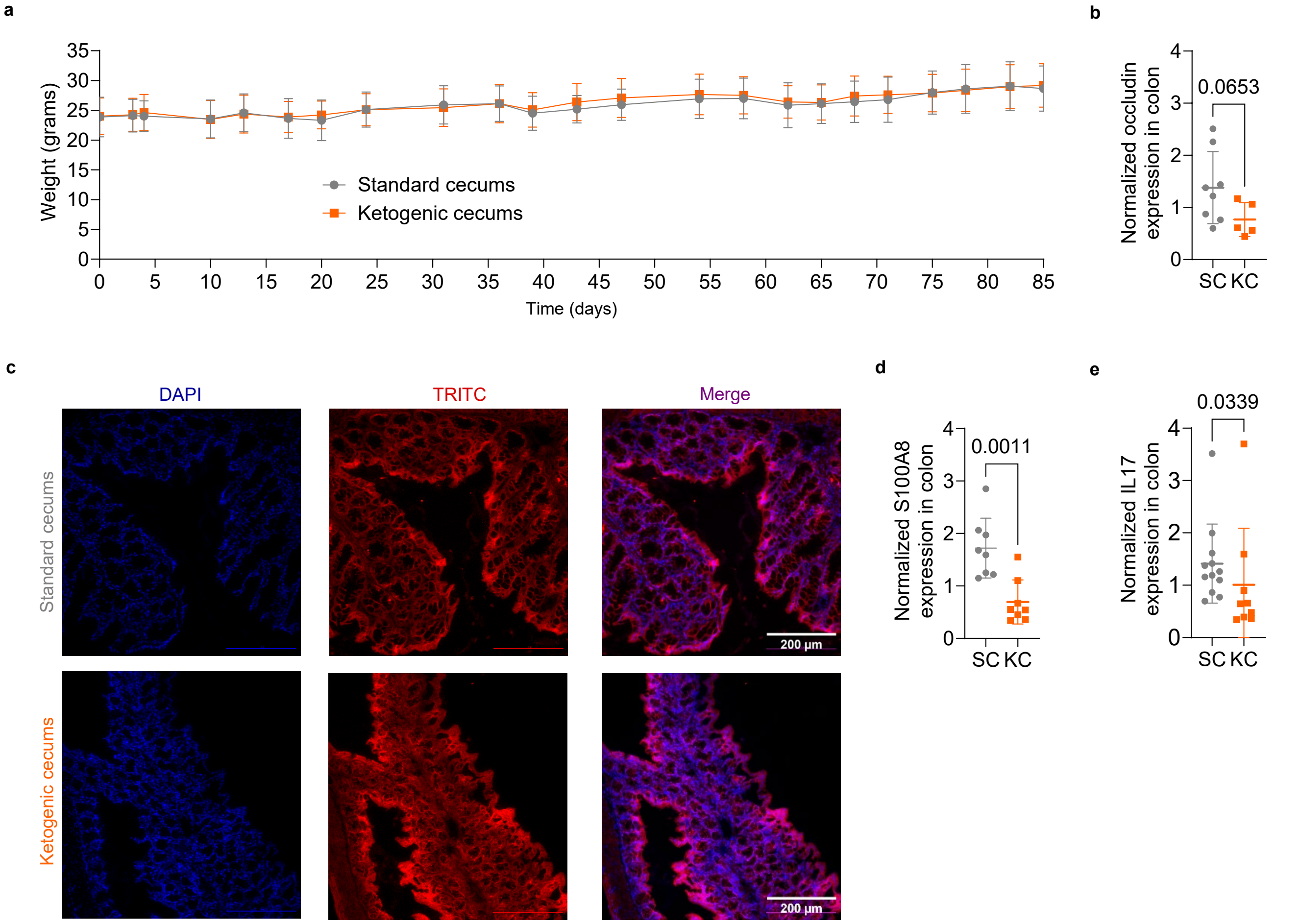

Supplementary Figure 6. | a. Mouse body weight (grams) over time (days) in the CMT experiment. **b-c.** Colonic occludin gene expression (**b**) and protein expression (**c**) in KC and SC mice, as measured by qPCR (**b**) and immunofluorescent staining (occludin-TRITC in red and DAPI nuclear stain in blue, one representative colon per condition is shown (two sections were stained per mouse (n=5 SD and n=5 KD mice) with similar results), scale bar = 200 µM, **c**). **d-e.** S100A8 (**e**) and IL-17 (**e**) gene expression in KC and SC mice in the CMT experiment, as measured by qPCR. Data is shown as mean±SD in (**a**), (**b**), (**d**) and (**e**). Two-tailed Mann-Whitney U test in (**b**), (**d**) and (**e**). Data in (**a**) shows n=16 mice per condition. Data in (**b**), (**d**) and (**e**) shows n=8 mice per condition. Source data are provided as a Source Data file.

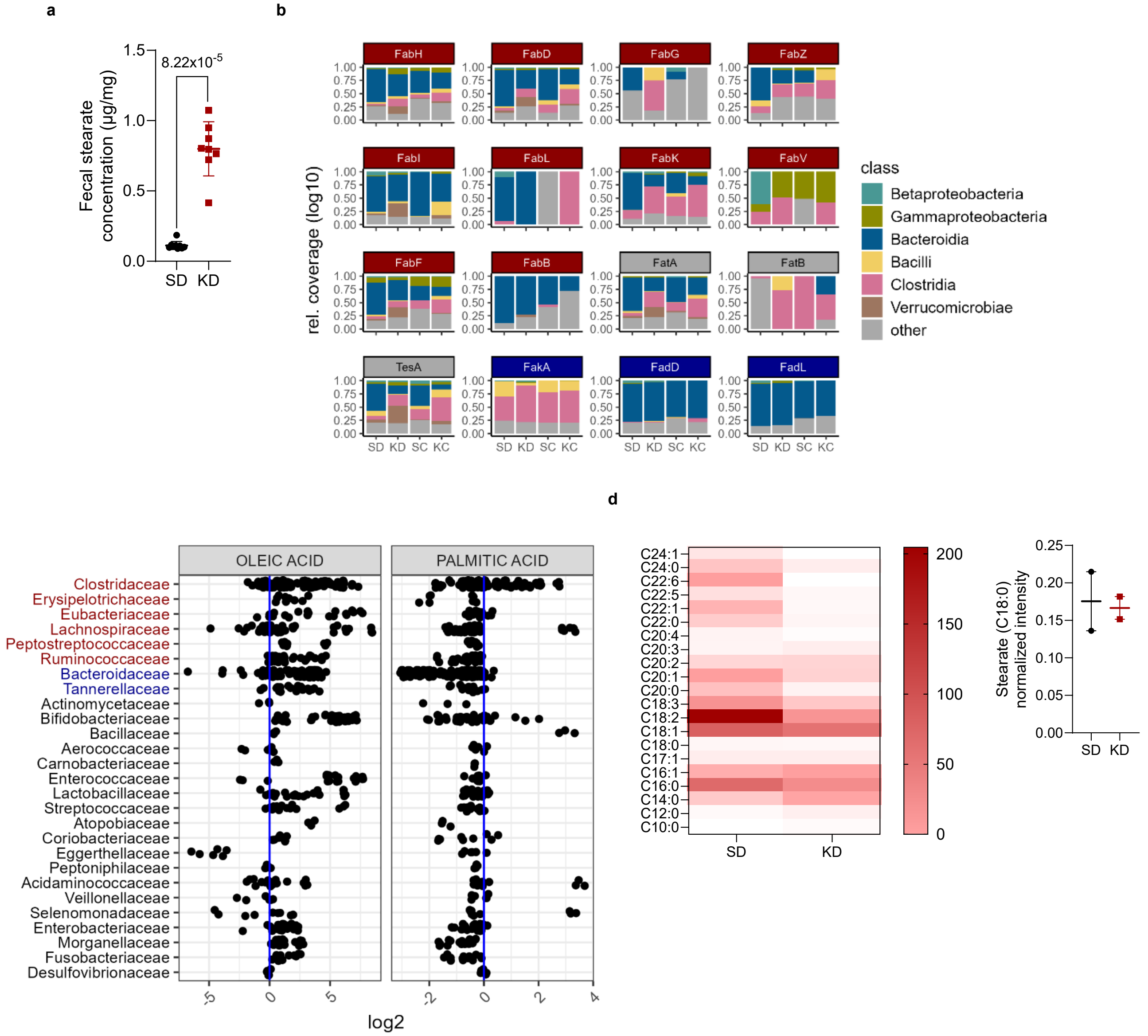

Supplementary Figure 7. | a. Stearic acid levels detected in fecal samples from SPF KD- and SD-fed mice (Fig. 4**c**) by GC-MS (µg/mg of feces in non-polar phase). Data is shown as mean±SD from n=9 KD mice and n=8 SD mice. **b.** Relative taxonomic assignment of contigs encoding for the selected KOs of interest at class level in fecal samples from KD- and SD-fed mice, and KC and SC recipient mice, at endpoint. Data shows n=8 SPF KD-fed mice and n=9 mice SPF SD-fed mice, pooled from two independent experiments, and n=8 KC and n=8 SC recipient mice from one experiment. Shading of genes in red indicates synthesis-related genes, shading in gray indicates acyl-CoA thioesterases and shading in blue indicates genes involved in acquisition of extracellular long chain fatty acids. **c.** Levels of oleic acid (**left**) and palmitic acid (**right**) measured in bacterial cultures (in relation to sterile growth medium, shown as log2) of the Sonnenburg dataset published by Han *et al*. Families of the class Clostridia are listed in red, while families of the class Bacteroidia are listed in blue. **d.** Free long-chain fatty acid profiles (**left,** heatmap shows means) and free stearic acid content detected in SD and KD (**right,** shown as mean±SD). Two technical replicates per diet are shown. Source data are provided as a Source Data file.

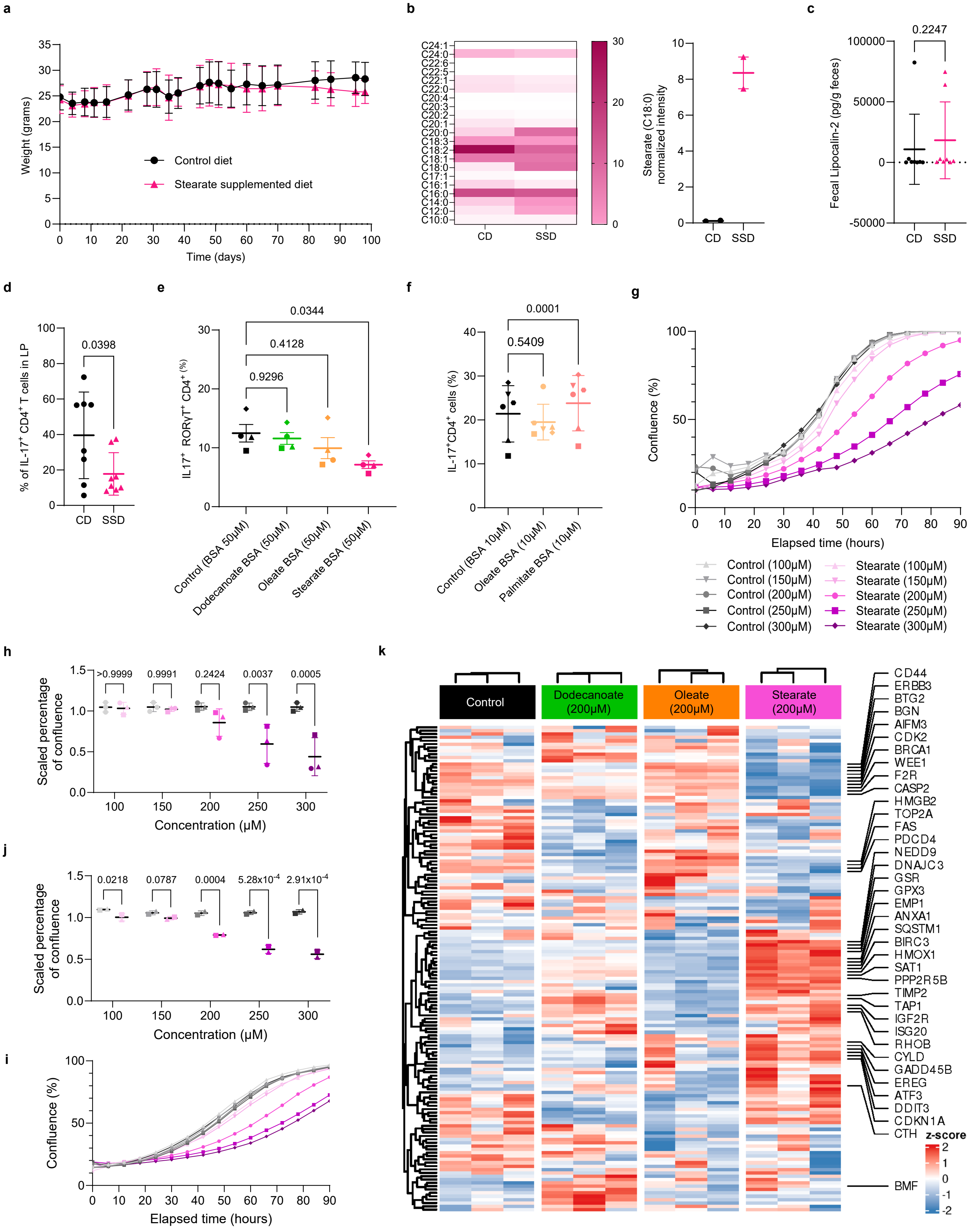

Supplementary Figure 8. | a. Mouse body weight (grams) over time (days) in stearate-supplemented diet-fed (SSD) mice and control-diet-fed (CD) mice. **b.** Free long-chain fatty acid profiles (**left,** heatmap shows means) and free stearic acid content detected in SD and KD (**right,** shown as mean±SD). Two technical replicates per diet are shown. **c.** Fecal lipocalin-2 levels (pg per gram of feces) in CD and SSD mice as detected by ELISA. **d.** Frequency of CD4+ IL-17+ cells in colonic lamina propria in CD and SSD mice, as detected by flow cytometry. **e-f.** Th17 differentiation upon treatment with BSA-complexed dodecanoic acid, oleic acid and stearic acid (50µM, **e**) and with BSA-complexed palmitic and oleic acid (10µM, **f**). Data is shown as mean±SD in (**a**), (**b, right**), (**c**), (**d**), (**e**) and (**f**). Data in (**a**), (**c**) and (**c**) shows n=8 mice per condition. Data in (**e**) shows n=4 and data in (**f**) shows n=6 biological replicates (mice) indicated by different datapoint shapes, from one experiment each. Unpaired Mann-Whitney U test in (**c**). Unpaired T test in (**d**), data passed Shapiro-Wilk normality test p = 0.29. Paired ordinary one-way ANOVA in (**e**) and (**e**). **g-j.** HCT116 (**g**) and Caco2 (**i**) proliferation over time and scaled HCT116 (**h**) and Caco2 (**j**) confluence at 72 hours after treatment with stearic acid or corresponding control (100µM, 150 µM, 200 µM, 250 µM, 300 µM). Data shows mean (**g**) and (**i**) or mean±SD (**h**) and (**j**) from n=3 independent experiments, indicated by different datapoint shapes, each with eight technical replicates per condition. Two-way ANOVA with multiple comparisons in (**h**) and (**h**). **k.** Heatmap showing the expression of apoptosis hallmark genesets in HT-29 cells treated with dodecanoic acid, oleic acid and stearic acid (200µM). Labeled genes are identified as being differentially expressed in the DGE analysis ($|\log_2 FC| > 0.25$ and p-adj < 0.05). Data shown in (**k**) is from n=3 independent experiments. Source data are provided as a Source Data file.

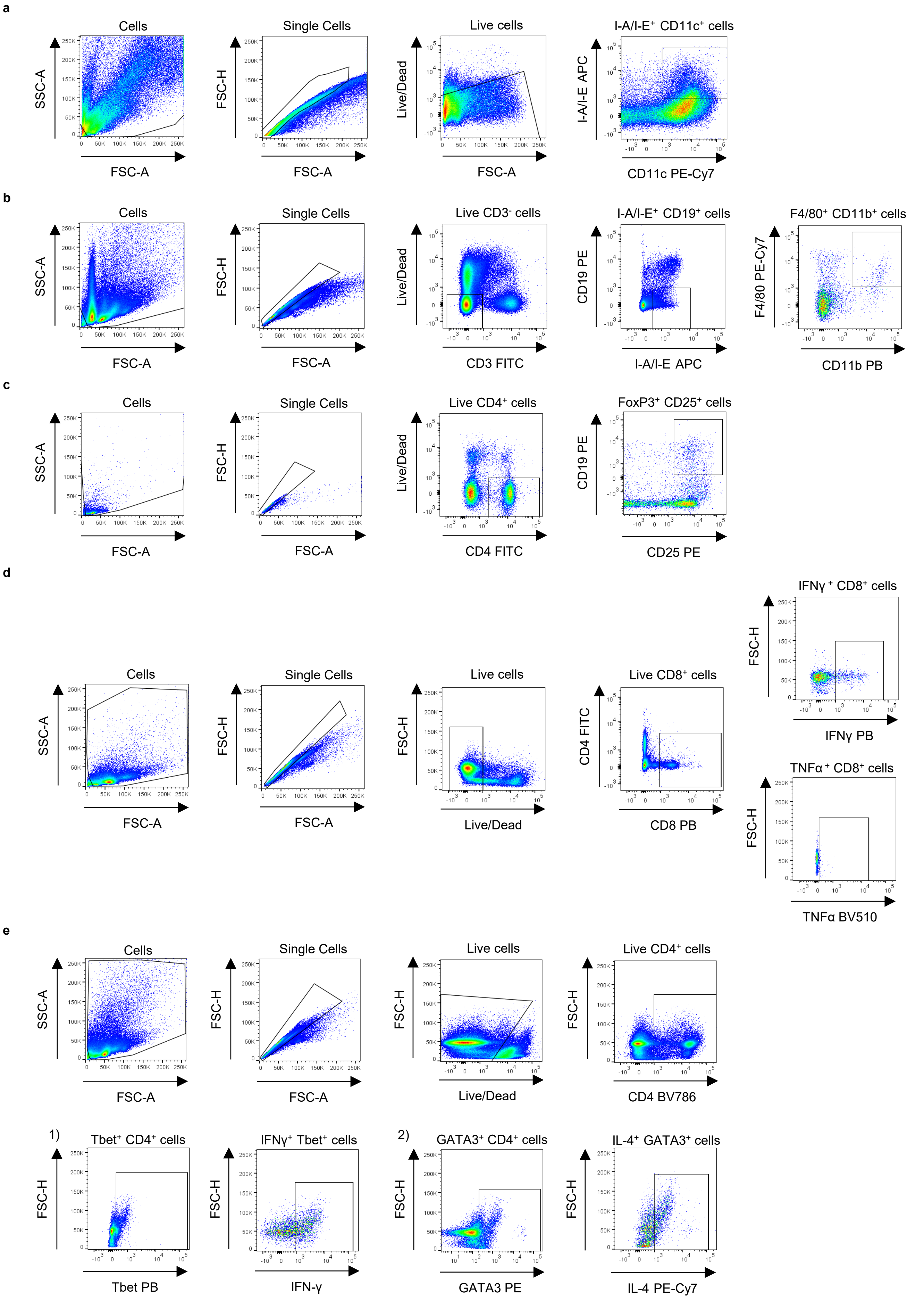

Supplementary Figure 9. | a. Representative gating strategy of dendritic cells. **b.** Representative gating strategy of macrophages. **c.** Representative gating strategy of regulatory T cells. **d.** Representative gating strategy of CD8+ T cells. **e.** Representative gating strategy of T helper 1 (subpanel 1) and T helper 2 cells (subpanel 2).

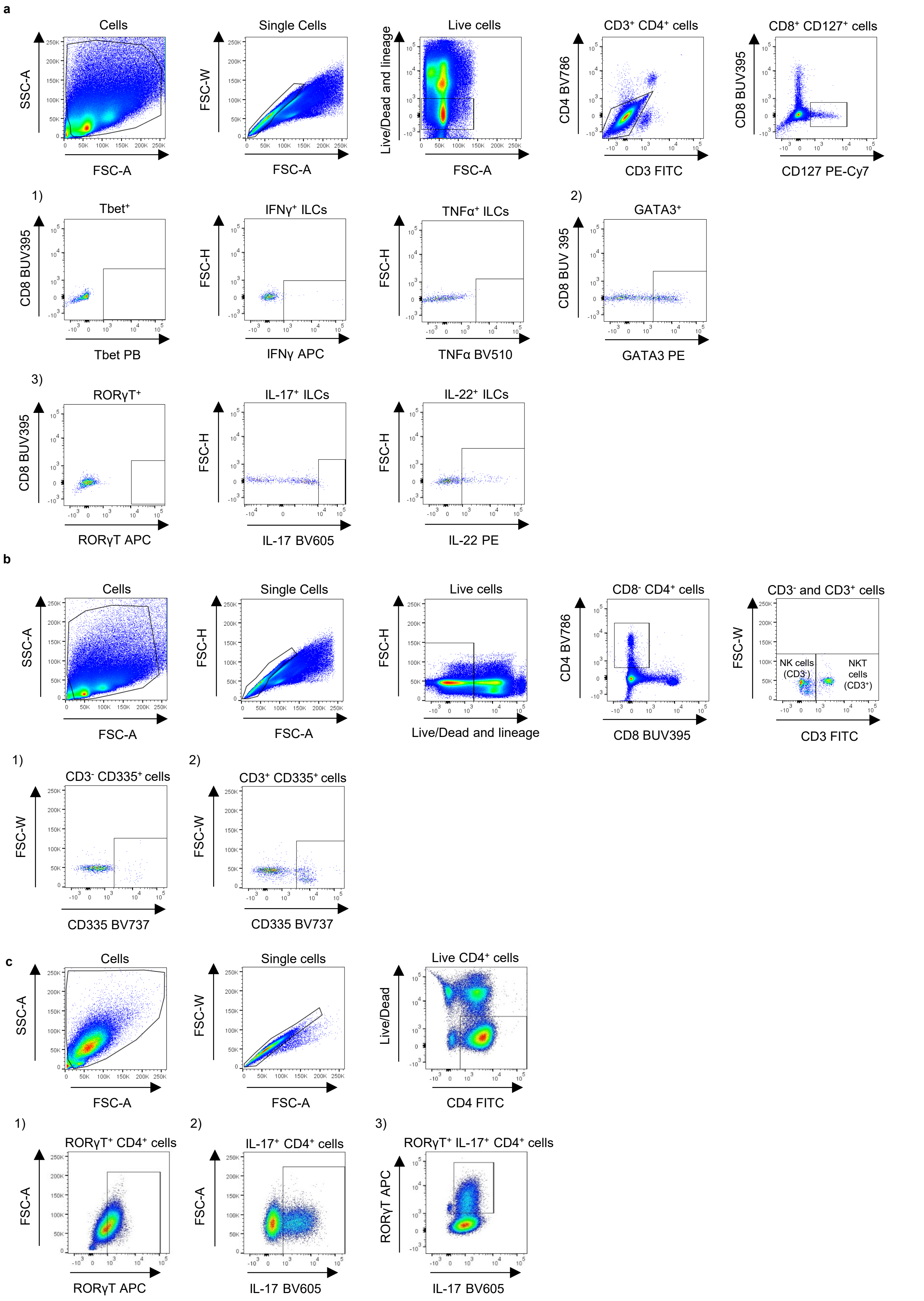

Supplementary Figure 10. | a. Representative gating strategy of innate lymphoid cells (ILC) - ILC1 (subpanel 1), ILC2 (subpanel 2), ILC3 (subpanel 3). Transcription factors and cytokines are stained in two separate batches. **b.** Representative gating strategy of natural killer (subpanel 1) and natural killer T (subpanel 2) cells. **c.** Representative gating strategy of T helper 17 cells – transcription-factor-based phenotyping (subpanel 1), cytokine-based phenotyping (subpanel 2) and the combined strategy (subpanel 3).

Detailed metabolomics workflows

Untargeted metabolomics of human fecal samples

Human fecal sample homogenization for metabolite extraction

Circa 1g of frozen human fecal material was placed in a microvial pre-cooled in liquid nitrogen. The samples were milled in a cryogenic grinder (6875D Freezer/Mill®, SPEX® SamplePrep, Instrument Solutions; four minutes of precooling, followed by three cycles of two-minute-long runtime at a rate of 12 cycles per second, spaced by one minute of cooling), reducing them to a fine powder. Upon removal from the grinder, the samples were maintained in liquid nitrogen, aliquoted and then stored at -80°C until processing. The samples were not thawed at any time during homogenization.

Metabolite extraction from human fecal samples for untargeted LC-MS (HILIC)

50mg of homogenized fecal powder was resuspended in 500µL of MilliQ® water and further homogenized at 6000rpm for 30 seconds at 4°C in a ThermoMixer. Samples were centrifuged at maximum speed for five minutes at 4°C. An internal standard mix (ISM) was prepared (1mg/mL of 6-chloropurine riboside (Sigma-Aldrich), 2-chloroquinoline-3-carboxylic acid (Sigma-Aldrich), 4-chloro-DL-phenylalanine (Sigma-Aldrich), Nε-trifluoroacetyl-L-lysine(Sigma-Aldrich), sucralose (Sigma-Aldrich), caffeine-trimethyl (Sigma-Aldrich) in MilliQ® water). Supernatants were collected and filled up to 75µL with MilliQ water, then 75µL of the ISM were added to each sample and they were vortexed thoroughly. Proteins were precipitated by the addition of 600µL of ACN (Carl ROTH) +1% formic acid (v/v, Sigma-Aldrich) at 4°C to 120µL of sample and then vortexed thoroughly. Samples were incubated for five minutes at 4°C in a ThermoMixer Comfort (Eppendorf), then centrifuged for five minutes at maximum speed at 4°C. Phospholipids were removed by transferring the supernatants to a phospholipid removal plate (Phree) and vacuum for 2-7 inches Hg was applied until the filtrate collected in the deep well plate. 500µL were transferred into 1.5mL Eppendorf tubes and the solvents were evaporated in a rotary vacuum evaporator

at 4°C overnight. Then, the temperature of the rotary vacuum evaporator was increased to room temperature for 25 minutes to avoid water condensation on the surface of the tube. Samples were stored at -80°C until LC-MS analysis was performed. An eluent was prepared (20mM ammonium acetate (AmAc, Sigma-Aldrich) in 90% ACN+ 0.1% formic acid). Samples were reconstituted in 50µL of a 5% solution of the eluent in MilliQ® water, then filtered through PHENEX-RC 4mm syringe filters (Phenomenex) into glass LC vials (Chromatographie Zubehör Trott) with microinserts (Chromatographie Zubehör Trott) and screwcaps (Chromatographie Zubehör Trott). 5µL per sample were pooled in a separate vial. Hydrophilic interaction liquid chromatography (HILIC) was performed using 20mM AmAc in water as mobile phase A and 20mM AmAc in 90% ACN as mobile phase B. SeQuant ZIC-pHILIC 150x2.1mm columns (Merck Millipore) were used and analysis was performed using a Q Exactive HF mass spectrometer (ThermoFisher Scientific).

LC-MS HILIC

For untargeted HILIC LC-MS/MS analysis, 3 µl of reconstituted sample were injected. Metabolites were separated at a flow rate of 200 µl/ml with the following gradient: 90 % B for 1.5 min, followed by a decrease to 20% B within 15 min, then 20% B for 2 min, increase to 90% B within 2 min, then 90% B for 13 min. The column temperature was set to 50°C. Samples were acquired using the following setting for full scan mode: Resolution - 120,000, m/z range - 60-900, AGC target - 1e6, maximum injection time – 70 ms. ddMS2 was applied using the following settings: Resolution - 30,000, AGC target - 5e5, maximum injection time – 70, topN - 5, Normalized collision energy – 20. Samples were acquired in positive and negative ionization mode separately.

Untargeted metabolomics and bile acid quantification of mouse plasma samples

Metabolite extraction from murine plasma samples for untargeted GC-MS

Plasma samples were thawed on ice and centrifuged at 15000g for three minutes at 4°C. An ISM was prepared (1mg/mL of ribitol, pentanedioic-d6 acid and tridecanoic-d25 acid in MilliQ® water). 14µL of the ISM was added to 35µL of plasma at 4°C and the samples were vortexed thoroughly. Proteins were precipitated by the addition of 160µL of methanol (Sigma-Aldrich) at 4°C and vortexed thoroughly. Samples were incubated for five minutes at 4°C, then centrifuged for five minutes at 4°C in a ThermoMixer Comfort (Eppendorf). 175µL of the supernatant was added to 140µL of chloroform (for HPLC, 99.9%, CHROMASOLV®, Sigma-Aldrich) at room temperature and vortexed thoroughly. 90µL of water were added to the samples, which were again vortexed thoroughly and incubated for 10 minutes at 4°C, then centrifuged at maximum speed for five minutes at 4°C in a ThermoMixer. 200µL of the upper (polar) phase was transferred into a GC vial (Chromatographie Zubehör Trott) with a microinsert (Chromatographie Zubehör Trott). Solvents were evaporated in a SpeedVac® (ThermoFisher Scientific) at 4°C overnight. Then, the temperature of the SpeedVac® was increased to room temperature for 25 minutes to avoid water condensation on the surface of the glass vial. Samples were capped and stored at -80°C until GC-MS analysis was performed. Metabolite derivatization was performed by using a multi-purpose sampler (Gerstel). Dried extracts were dissolved in 20µL pyridine (≥99.5% for GC, Sigma-Aldrich), containing 20mg/mL methoxyamine hydrochloride (for GC derivatization LiChropur™, 97.5-102.5%, Sigma-Aldrich), at 45°C for 120 min under shaking. After adding 20µL of N-trimethylsilyl-N-methyl trifluoroacetamide (MSTFA, Machery-Naegel), samples were incubated at 45°C for 30 minutes under continuous shaking. GC-MS analysis was performed using an 7890B GC (Agilent Technologies) coupled to a 5977A mass selective detector (MSD, Agilent Technologies).

Upper phase on GC3 – SCAN plasma

A sample volume of 1 µL was injected into a Split/Splitless inlet, operating in split mode (10:1) at 270 °C. The gas chromatograph was equipped with a 5 m guard column + 30 m (I.D. 250 µm, film 0.25 µm) DB-35MS capillary column (Agilent J&W GC Column). Helium was used as the carrier gas with a constant flow rate of 1.2 mL/min. The GC oven temperature was held at 90 °C for 1 min and increased to 270 °C at 20 °C/min. Then, the temperature was increased to 325 °C and held for 5 min. The total run time was 15 min. The transfer line temperature was set constantly to 280 °C. The mass selective detector (MSD) was operating under electron ionisation at 70 eV. The MS source was held at 230 °C and the quadrupole at 150 °C. Full scan mass spectra were acquired from m/z 70 to m/z 600. All GC-MS chromatograms were processed using the MetaboliteDetector software.

Metabolite extraction from murine plasma samples for untargeted LC-MS (HILIC)

Plasma samples were thawed on ice and centrifuged at 15000g for three minutes at 4°C. An ISM was prepared (1mg/mL of 6-chloropurine riboside, 2-chloroquinoline-3-carboxylic acid, 4-chloro-DL-phenylalanine, Nε-trifluoroacetyl-L-lysine, sucralose, caffeine-trimethyl in MilliQ® water). 20µL of the ISM was added to 20µL of plasma at 4°C and the samples were vortexed thoroughly. Proteins were precipitated by the addition of 151µL of methanol to 38µL of sample at 4°C and vortexed thoroughly. Samples were incubated for 15 minutes at -20°C, then centrifuged for five minutes at 4°C in a ThermoMixer. Phospholipids were removed by diluting samples in 300µL of methanol, then transferring them to a Phree phospholipid removal plate and vacuum for 2-7 inches Hg was applied until the filtrate collected in the deep well plate. 350µL were transferred into 1.5mL Eppendorf tubes and the solvents were evaporated in a SpeedVac® at 4°C overnight. Then, the temperature of the SpeedVac® was increased to room temperature for 25 minutes to avoid water condensation on the surface of the tube. Samples were stored at -80°C until LC-MS analysis was performed. An eluent was prepared (20mM AmAc in 90% ACN + 0.1% formic acid). Samples were reconstituted in 50µL of a 5% solution of the eluent in MilliQ®

water, then filtered through PHENEX-RC4mm syringe filters into glass LC vials with microinserts and screwcaps. HILIC was performed using 20mM AmAc in water as mobile phase A and 20mM AmAc in 90% ACN, 50:50 (v/v) + 0.1% formic acid as mobile phase B. SeQuant ZIC-pHILIC 5 µm polymeric sorbent columns were used and analysis was performed using a Thermo Q Exactive HF mass spectrometer.

Murine plasma BA quantification by ultra-high performance liquid chromatography – tandem mass spectrometry (UHPLC-MS/MS)

10 µL of plasma were used for bile acid (BA) quantification. BAs were analysed using UHPLC-MS/MS, consisting of an ExionLC (Sciex) coupled to a QTrap 5500 mass spectrometer (Sciex). Electrospray ionization was performed in the negative ionization mode. Chromatographic separation was run on an ethylene bridged hybrid (BEH) C18 column (2.1x100 mm, 1.7µm, Waters). The mobile phase consisted of water, containing 0.1% formic acid and 5mM ammonium acetate or acetonitrile (ACN, Carl ROTH). The analytes were separated by a gradient elution. BA detection was performed in the multiple reaction monitoring (MRM) mode. Cholic acid (CA), hyocholic acid (HCA), chenodeoxycholic acid (CDCA), deoxycholic acid (DCA), 7-oxo-deoxycholic acid (7-oxo-DCA), murideoxycholic acid (MDCA), lithocholic acid (LCA), ursodeoxycholic acid (UDCA), hyodeoxycholic acid (HDCA), muricholic acids (α-MCA, β-MCA and ω-MCA), taurocholic acid (TCA), taurohyocholic acid (THCA), taurochenodeoxycholic acid (TCDCA), taurodeoxycholic acid (TDCA), taurolithocholic acid (TLCA), tauroursodeoxycholic acid (TUDCA), taurohyodeoxycholic acid (THDCA), tauro-α-muricholic acid (T-α-MCA), tauro-β-muricholic acid (T-β-MCA) and tauro-ω-muricholic acid (T-ω-MCA) standard substances, as well as the deuterated BA internal standard (IS) substances d4-CA, d4-GCA and d4-TCA were purchased from Sigma-Aldrich Chemie GmbH (Taufkirchen, Germany), Avanti Polar Lipid (Alabaster, USA) and Steraloids (Newport, USA). Further details can be found in García-Cañaveras et al., 2012[2].

Untargeted metabolomics and short chain fatty acid quantification of mouse fecal samples

Mouse fecal pellet homogenization for metabolite extraction

Mouse fecal pellets were placed in 0.5mL Precellys® tubes (VWR) containing five ceramic beads each and MilliQ® water was added to each sample at a 1:16 dry weight to water ratio. The samples were homogenized at 6000rpm, for two 30-second-long cycles at 4°C, in a Precellys®24 Homogenizer (Bertin Corp.). Samples were then incubated at 4°C for ten minutes, then centrifuged at maximum speed for 10 minutes at 4°C. Samples were maintained on ice and in the dark. The supernatant was used for further downstream processing (metabolite extraction for GC-MS and LC-MS analyses, see below for GC-MS polar and above for HILIC).

Metabolite extraction from murine fecal samples for short-chain fatty acid (SCFA) quantification

For targeted and absolute quantitative SCFA detection in mouse fecal samples, derivatization was performed in the organic phase using diethyl ether (VWR). A dilution series of a volatile free acid mix (VFAM, Supelco®, Sigma-Aldrich) was prepared in MilliQ® water (final concentrations of 0µM, 10µM, 50µM, 250µM, 500µM, 750µM, 1000µM, 2000µM, 3000µM and 4000µM). Quality control samples were prepared independently of the calibration curve (final concentrations 15µM, 75µM, 80µM and 2750µM of VFAM in MilliQ® water). A stock solution of internal standard (IS) was prepared by adding 253µL of 2-ethylbutyric acid (Sigma-Aldrich) to 747µL of methanol, and further diluting it 1:100 in MilliQ® water (final stock concentration 20mM). Then 10µl of the IS (final concentration 2mM) and 10µl of 37% hydrochloric acid (Sigma-Aldrich) were added to 180µl of fecal supernatant fluid, of extraction blank (180µL of MilliQ® water), of the dilution series of VFAM and of the quality control samples. After 15 minutes of agitation at 15°C in the Thermomixer (Eppendorf), 1 ml of 99% diethyl ether was added to each sample. The samples were again agitated as previously described, then centrifuged for five minutes at maximum speed at 15°C. The upper phase (900µL) was transferred into a fresh 2mL Eppendorf tube. Then 1 ml of diethyl ether was

once again added to the tube containing the lower phase and a second agitation, centrifugation and transfer of the upper phase were performed, resulting in 1800µL of upper phase per sample. The samples were then aliquoted in triplicates into GC vials (250µl per replicate), and 25µl of N-Methyl-N-(tert-butyldimethylsilyltrifluoro acetamide) w/1% tert butyldimethylchlorosilane (MTBSTFA, Interscience) were added. The samples were analyzed on an 8890 GC (Agilent Technologies), coupled to an 5977B MSD (Agilent Technologies). A sample volume of 1 µL was injected into a Split/Splitless inlet, operating in split mode (20:1) at 280 °C. The gas chromatograph was equipped with a 30 m (I.D. 250 µm, film 0.25 µm) ZB-5MSplus capillary column (Phenomenex) with 5 m Guardian in front of the analytical column. Helium was used as carrier gas with a constant flow rate of 1.4 mL/min.

The GC oven temperature was held at 80 °C for 1 min and increased to 170 °C at 10 °C/min. Then, the temperature was increased to 280 °C and held for 5 min (post run time). The total run time was 15 min. The transfer line temperature was set to 280 °C. The mass selective (MS) detector was operating under electron ionization at 70 eV. The MS source was held at 230 °C and the quadrupole at 150 °C. The detector was switched off during elution of MTBSTFA. For precise quantification, GC-MS measurements of target analytes were performed in selected ion monitoring mode using defined quantifier and qualifier ions (dwell times: 20 ms). Mass spectrometric data was acquired with MassHunter GC/MS Data Acquisition software (Version 10.0, Build 10.0.384.1).

Table 1. Selected quantifer and qualifier ions for short chain fatty acids.

**Analyte Name**	**Quantification Ion (*m/z*)**	**Qualification Ion I (m/z)**	**Qualification Ion II (m/z)**
Formic acid 1TBDMS	103	75	99
Acetic acid 1TBDMS	117	75	99
Propionic acid 1TBDMS	131.1	75	115
Isobutyric acid 1TBDMS	145.1	75	115

Butyric acid 1TBDMS	145.1	75	115
Isovaleric acid 1TBDMS	159.1	75	201.1
Valeric acid 1TBDMS	159.1	75	201.1
IS 2-Ethylbutyric acid 1TBDMS	173.1	75	115
4-Methylvaleric acid 1TBDMS	173.1	75	215.1
Hexanoic acid 1TBDMS	173.1	75	131
Heptanoic acid 1TBDMS	187.1	75	131

Data analysis was performed in the Agilent MassHunter Quantitative Analysis for GCMS software (Version 10.2, Build 10.2.733.8). Absolute quantification was achieved by using external calibration curves (Volatile Free Acid Mix, Sigma-Aldrich; calibrated range 15 to 2750 µmol/L) and the response ratio of all target compounds.

Metabolite extraction from murine fecal samples for untargeted GC-MS and LC-MS

An ISM was prepared (2µg/mL of ribitol, pentanedioic-d6 acid and d-mannose and 10µg/mL tridecanoic-d25 acid, 6-chloropurine riboside, 4-chloro-DL-phenylalanine, Nε-trifluoroacetyl-L-lysine and thionicotinamide adenine dinucleotide (Sigma-Aldrich) in MilliQ® water). 40µL of the ISM was added to 100µL of the fecal supernatant fluid. 80µL of this mixture was added to 320µL of methanol, vortexed thoroughly, incubated for five minutes at 4°C at maximum speed in an Eppendorf ThermoMixer, and then centrifugated for five minutes at 4°C at maximum speed. 350µL of supernatant were added to 280µL of chloroform. 180µL of MilliQ® water were added, the samples were vortexed thoroughly, incubated for ten minutes at 4°C at maximum speed in an ThermoMixer (Eppendorf), then centrifugated for five minutes at 4°C at maximum speed. The extract was then split – 200µL of the upper (polar) phase were aliquoted into a GC vial with a micro-insert and the rest of the upper (polar) phase was filtered through a PHENEX-

RC syringe filter (Phenomenex), and 200µL were retained in Eppendorf tubes for further processing. 200 µl of the lower (non-polar) phase were aliquoted into a GC vial with micro-insert. The solvents were evaporated in a SpeedVac® at -4°C overnight. Then, the temperature of the SpeedVac® was increased to 25°C for 25 minutes to avoid water condensation on the surface of the glass vial. Samples were protected from light exposure and stored at -80°C. Samples for GC-MS (polar and non-polar) were analyzed on an 7890B GC (Agilent Technologies), coupled to an 5977A MSD (Agilent Technologies). Samples for LC-MS analysis (polar) were reconstituted in 80µL 50% ACN in water, transferred into LC vials for LC-MS analysis and analyzed on a Vanquish UHPLC (ThermoFisher Scientific), coupled to a Q Exactive HF mass spectrometer (ThermoFisher Scientific).

GC-MS polar (MeOX120min TMS30min SPLITLESS 30min.M)

Metabolite derivatization was performed by using a multi-purpose sampler (Gerstel). Dried extracts were dissolved in 20µL pyridine (≥99.5% for GC, Sigma-Aldrich), containing 20mg/mL methoxyamine hydrochloride (for GC derivatization LiChropur™, 97.5-102.5%, Sigma-Aldrich), at 45°C for 120 min under shaking. After adding 20µL of N-trimethylsilyl-N-methyl trifluoroacetamide (MSTFA, Machery-Naegel), samples were incubated at 45°C for 30 minutes under continuous shaking. A sample volume of 1 µL was injected into a Split/Splitless inlet, operating in split mode (10:1) at 270 °C. The gas chromatograph was equipped with a 5 m guard column + 30 m (I.D. 250 µm, film 0.25 µm) DB-35MS capillary column (Agilent J&W GC Column). Helium was used as the carrier gas with a constant flow rate of 1.2 mL/min. The GC oven temperature was held at 90 °C for 1 min and increased to 270 °C at 9 °C/min. Then, the temperature was increased to 320 °C at 25°C/min and held for 7 min. The total run time was 30 min. The transfer line temperature was set constantly to 280 °C. The mass selective detector (MSD) was operating under electron ionisation at 70 eV. The MS source was held at 230 °C and the quadrupole at

150 °C. Full scan mass spectra were acquired from m/z 70 to m/z 700. All GC-MS chromatograms were processed using the MetaboliteDetector software.

GC-MS unpolar (OD 30uLTBDMS60min SP10 35min NP SCAN.M)

Metabolite derivatization was performed by using a multi-purpose sampler (Gerstel). After adding 30µL of N-Methyl-N-(tert-butyldimethylsilyltrifluoro acetamide) w/1% tert butyldimethylchlorosilane (MTBSTFA, Interscience), samples were incubated at 55°C for 60 minutes under continuous shaking. A sample volume of 1 µL was injected into a Split/Splitless inlet, operating in split mode (10:1) at 280 °C. The gas chromatograph was equipped with a 30 m (I.D. 250 µm, film 0.25 µm) Zebron ZB-35 capillary column (Phenomenex). Helium was used as the carrier gas with a constant flow rate of 1.2 mL/min. The GC oven temperature was held at 100 °C for 1 min and increased to 325 °C at 7.5 °C/min. Then, the temperature held for 4 min. The total run time was 35 min. The transfer line temperature was set constantly to 280 °C. The mass selective detector (MSD) was operating under electron ionisation at 70 eV. The MS source was held at 230 °C and the quadrupole at 150 °C. Full scan mass spectra were acquired from m/z 70 to m/z 700. All GC-MS chromatograms were processed using the MetaboliteDetector software.

LC-MS HILIC

(see above)

Pre- and post-processing of untargeted metabolomics data

Metabolomic profiling data pre-processing of untargeted LC-MS datasets

Raw data files obtained through LC-MS of human stool and murine plasma and stool samples were processed in TraceFinder (version 5.1.203.0) for peak identification and annotation. Three different in-house libraries, generated with reference standards, as well as one commercially available library, mzCloud Offline for mzVault 2.3_Omics_2020A.db, in the Advanced Mass Spectral Database (AMSD) (HighChem, from ThermoFischer Scientific), were used for peak annotation, with the adduct formulas $[M+H]^+$ and $[M-H]^-$. Annotated features and integration tables were exported for post-processing.

Metabolomic profiling data pre-processing of untargeted GC-MS datasets

Raw data files obtained through GC-MS murine plasma and stool samples were processed in MetaboliteDetector[77] (version 3. 220190704) for peak identification and annotation. An in-house library, generated with reference standards was used for peak annotation. Deconvolution settings were applied as follows: peak threshold = 5, minimum peak height = 5, bins per scan = 10, deconvolution width = 5 scans, no baseline adjustment, minimum 15 peaks per spectrum and no minimum required base peak intensity. The data was normalized using the response ratio of the integrated peak area of each metabolite and the integrated peak area of the IS, as described in[78]. Annotated features and integration tables were exported for post-processing.

Metabolomic profiling post-processing data analysis

For murine plasma metabolites identified in the SPF experiment (Extended Data Fig. 4), data was manually curated based on the pooled sample, spiked with the ISM. Features, which could not be confirmed through MS2 data (from the commercially available database) or through the ISM were filtered out. Metabolites with missing (N/A) values were also filtered out. Single metabolite intensities were

normalized to the total metabolite area and corrected using the mean value of total metabolite values from all samples. LC-MS and GC-MS data were unified, and duplicate metabolites were filtered out, based on reliability and consistency of the peak areas.

Human stool sample data was similarly processed (but without the element of data unification between methods).

For murine plasma metabolites identified in the GF experiment (GC-MS), metabolites with missing (N/A) values were filtered out.

Murine stool SCFA content was quantified based on an IS and using the MassHunter Quantitative Analysis Software (version 10.2.733.8). In the LC-MS and GC-MS datasets, metabolites with over 20% missing (N/A) values were filtered out. Data from murine stool untargeted LC-MS, GC-MS and SCFA quantification was unified and duplicate metabolites were filtered out, based on reliability and consistency of the peak areas.

The deuterium labelled tridecanoic acid (C13:0-d25, Eurisotop D-4002) was used as internal standards to enable absolute quantification of stearic acid (C18:0) in the non-polar phase of fecal samples analyzed by GC-MS (linked to Extended Fig. 7a).

Long chain fatty acid quantification from mouse fecal and plasma samples and mouse diet

Extraction and LC-MS measurement of fatty acids -Total fatty acid extraction from mouse plasma

8 µl of mouse plasma were mixed with 392 µl ACN/H_2O 4+1 + internal standards (IS) (Tridecanoic acid-d25 (D-4002), Methyl heptadecanoate-d33 (00889-10MG), Docosanoic Acid-d43 (D-4005), Stearic acid-d5 (DLM-2712-0.1), final concentration 2 µg/ml) and 50 µl 5 M HCl in a glass vial and incubated for 1 h at 100°C for acidic hydrolysis of lipids. Free fatty acids were extracted using 800 µl of methyl-tert-butyl ether (MTBE) and phase separation was induced by the addition of 400 µl H_2O. After centrifugation for 5 min at 21,000xg, 900 µl of the upper phase were transferred into a new glass vial and washed with 800 µl of H_2O. After centrifugation, 500 µl of the upper (non-polar) phase were transferred into a new glass vial and solvents were evaporated in a CentriVap® (Labconco) at -4°C overnight. Prior LC-MS measurement, dried total fatty acid extracts were reconstituted in 100 µl reconstitution solution (40% mobile phase B: 10 mM Ammonium Acetate in 88/10/2 isopropanol/acetonitrile/H2O and 60% mobile phase A: 10 mM Ammonium Acetate in 40/60 Acetonitrile/H2O + 100 ng/ml CUDA (10007923)) and filtered using Phenex-PTFE syringe filters.

Extraction and LC-MS measurement of fatty acids -Free fatty acid extraction from mouse fecal samples

Mouse fecal samples were placed in 2 mL Precellys® tubes (VWR) containing 600 mg ceramic beads and mixed with MeOH + IS (Tridecanoic acid-d25 (D-4002), Docosanoic Acid-d43 (D-4005), Stearic acid-d5 (DLM-2712-0.1), final concentration 2 µg/ml) in a sample weight to solvent ratio of 1:30. Samples were homogenized at 6000 rpm, for two 30-second-long cycles at 4°C, in a Precellys®24 Homogenizer (Bertin Corp.). After addition of MTBE (ratio MeOH/MTBE 3+10), samples were incubated at room temperature under continuous shaking at 2000 rpm for 30 min. The volume reflecting the equivalent amount of 7.5 mg fecal samples was transferred to a glass vial (975 µl). To increase the total volume, 325 µl MeOH/MTBE 3+10 were added and 250 µl H_2O were used to induce phase separation (ratio

MeOH/MTBE/H_2O 3/10/2.5). After mixing the samples for 3 min at room temperature and 1500 rpm, samples were centrifuged for 5 min at 2,500 xg and 500 µl of the upper (non-polar)_phase were transferred to a new glass vial and solvents were evaporated in a CentriVap® (Labconco) at -4°C overnight. Prior LC-MS measurement, dried free fatty acid extracts were reconstituted in 100 µl reconstitution solution (40% mobile phase B: 10 mM Ammonium Acetate in 88/10/2 isopropanol/acetonitrile/H2O and 60% mobile phase A: 10 mM Ammonium Acetate in 40/60 Acetonitrile/H2O + 100 ng/ml CUDA (10007923)) and filtered using Phenex-PTFE syringe filters.

Free and total fatty acid extraction from mouse diet

Mouse diet samples were placed in 7 mL Precellys® tubes (VWR) containing 10 steel beads and homogenized at 9000 rpm for 3 cycles of 30 seconds at 4°C to generate powders. Mouse diet powders were weighted and placed into 2 mL Precellys® tubes (VWR) containing 600 mg ceramic beads and mixed with MeOH + IS (Tridecanoic acid-d25 (D-4002), Methyl heptadecanoate-d33 (00889-10MG), Docosanoic Acid-d43 (D-4005), Stearic acid-d5 (DLM-2712-0.1), final concentration 2 µg/ml) in a sample weight to solvent ratio of 1:30. Samples were further homogenized at 6000 rpm, for two 30-second-long cycles at 4°C, in a Precellys®24 Homogenizer (Bertin Corp.). After addition of MTBE (ratio MeOH/MTBE 3+10), samples were incubated at room temperature under continuous shaking at 2000 rpm for 30 min. The volume reflecting the equivalent amount of 6 mg diet sample was transferred to a glass vial (780 µl). To increase the total volume, 520 µl MeOH/MTBE 3+10 were added and 250 µl H_2O were used to induce phase separation (ratio MeOH/MTBE/H_2O 3/10/2.5). After mixing the samples for 3 min at room temperature and 1500 rpm, samples were centrifuged for 5 min at 2,500 xg. 500 µl of the upper (non-polar) phase were transferred to a new glass vial for the investigation of free fatty acids and 200 µl, respectively, for the investigation of total fatty acids and solvents were evaporated in a CentriVap® (Labconco) at -4°C overnight. Prior LC-MS measurement, dried free fatty acid extracts were reconstituted

in 100 µl reconstitution solution (40% mobile phase B: 10 mM Ammonium Acetate in 88/10/2 isopropanol/acetonitrile/H2O and 60% mobile phase A: 10 mM Ammonium Acetate in 40/60 Acetonitrile/H2O + 100 ng/ml CUDA (10007923)) and filtered using Phenex-PTFE syringe filters.

For total fatty acid analysis, dried lipid extracts were dissolved in 120 µl of isopropanol and 250 µl 0.4 M NaOH was added to perform alkaline hydrolysis. After incubation for 30 min at 37°C, samples were acidified with 300 µl 2 M HCl and fatty acids were extracted using 500 µl hexane. After rigorous vortexing and centrifugation for 5 min at 2,500 xg, 400 µl of the upper phase was collected in a new glass vial and solvents were evaporated in a CentriVap® (Labconco) at -4°C. Prior LC-MS measurement, dried total fatty acid extracts were reconstituted in 100 µl reconstitution solution (40% mobile phase B: 10 mM Ammonium Acetate in 88/10/2 isopropanol/acetonitrile/H2O and 60% mobile phase A: 10 mM Ammonium Acetate in 40/60 Acetonitrile/H2O + 100 ng/ml CUDA (10007923)) and filtered using Phenex-PTFE syringe filters.

LC-MS analysis of fatty acids

Relative quantification of target compounds was performed using an Agilent 1290 LC coupled to an Agilent 6560 Q-TOF MS system equipped with a Dual Agilent Jet Stream ESI source. The column (Waters ACQUITY UPLC BEH C18, 150 x 2.1 mm, 1.7 µm particle size) used in this study was maintained at 45 °C. The autosampler was kept at 4 °C and the injection volume was 1 µL. The flow rate was set to 0.2 mL/min and the mobile phases consisted of 10 mM ammonium acetate in 60/40 H_2O/ACN (Eluent A, pH unadjusted) and 10 mM ammonium acetate in 88/10/2 IPA/ACN/H_2O (Eluent B, pH unadjusted).

The run consisted of a linear gradient from 40% to 100% Eluent B over 10 min, followed by an isocratic delivery of 100% Eluent B over 3 min, returning to 40 % B within 0.1 min and end with isocratic delivery of 40% Eluent B for 6.9 min. Total run time was 20 min per sample.

MS measurements were performed using electrospray ionization in negative mode (-ESI) with a capillary

voltage of 3500 V and a nozzle voltage of 0 V. The deprotonated target molecules were monitored in high resolution mode (slicer position: 5) and Extended Dynamic Range (2GHz) with the following Q-TOF MS conditions: drying gas temperature: 325 °C, drying gas flow: 10 L/min (nitrogen), nebulizer: 35 psi, sheath gas temperature: 350 °C, sheath gas flow: 12 L/min, fragmentor: 390 V, Oct RF Vpp: 600 V. Full scan spectra were acquired from *m/z* 50 to 1600 (2 spectra/sec). External mass calibration was performed before measurement of each set of samples. A reference solution (m/z 112.9855 and m/z 1033.9881) was used online mass correction during the acquisition. All data were acquired with Agilent Mass Hunter LC/MS Data Acquisition (ver B.09.00, Build 9.0.9044.0) and analyzed with Agilent Mass Hunter Profinder (ver 10.0 SP1, Build 10.0.10142.1). Target compounds were identified by exact mass (mass error ±5 ppm), isotopic pattern and retention time (±0.15 min) matching (Batch Targeted Feature Extraction). Semi-quantification was based on integrated peak area of the deprotonated target compound and normalization to internal standards. The deuterium labelled stearic acid (C18:0-d5, Eurisotop DLM-2712, 2 µg/ml in the extraction fluid) was used as internal standards to enable absolute quantification of stearic acid (C18:0) (linked to Figure 6c-d).
