# Supplementary material for: Ketogenic diet suppresses colorectal cancer through the gut microbiome long chain fatty acid stearate
Source: Nat Commun. 2025 Feb 20;16:1792. doi: 10.1038/s41467-025-56678-0 (PMC11842570; doi:10.1038/s41467-025-56678-0)
Supplement: Supplementary file 2 — Description of Additional Supplementary Files [file 41467_2025_56678_MOESM2_ESM.pdf]

Description of Additional Supplementary Files

File Name: Supplementary Data 1
Description: Metabolites detected in plasma from mice fed a ketogenic diet and a standard diet in an inflammatory model of CRC (GF)

File Name: Supplementary Data 2
Description: Metabolites detected in plasma from mice fed a ketogenic diet and a standard diet in an inflammatory model of CRC (SPF)

File Name: Supplementary Data 3
Description: Differentially abundant bacterial families detected over time in fecal samples from mice fed a ketogenic diet and a standard diet in an inflammatory model of CRC (GF)

File Name: Supplementary Data 4
Description: Differentially abundant bacterial families detected over time in fecal samples from mice fed a ketogenic diet and a standard diet in an inflammatory model of CRC (SPF)

File Name: Supplementary Data 5
Description: Metabolites detected in fecal samples from mice fed a ketogenic diet and a standard diet in an inflammatory model of CRC (SPF)

File Name: Supplementary Data 6
Description: Metabolites detected in fecal samples from recipient mice receiving FMT from donor mice fed a ketogenic diet and a standard diet in an inflammatory model of CRC

File Name: Supplementary Data 7
Description: Long-chain fatty acids detected in serum samples from mice fed a stearate-supplemented diet in an inflammatory model of CRC

File Name: Supplementary Data 8
Description: Long-chain fatty acids detected in fecal samples from mice fed a stearate-supplemented diet in an inflammatory model of CRC

File Name: Supplementary Data 9
Description: In vivo experimental workflows

File Name: Supplementary Data 10
Description: Antibodies

File Name: Supplementary Data 11
Description: Primers
