# Supplementary material for: Ketogenic diet suppresses colorectal cancer through the gut microbiome long chain fatty acid stearate
Source: Nat Commun. 2025 Feb 20;16:1792. doi: 10.1038/s41467-025-56678-0 (PMC11842570; doi:10.1038/s41467-025-56678-0)
Supplement: Supplementary file 14 — Reporting Summary [file 41467_2025_56678_MOESM14_ESM.pdf]

nature portfolio

Corresponding author(s): Elisabeth Letellier

Last updated by author(s): 27.01.2025

Reporting Summary

Nature Portfolio wishes to improve the reproducibility of the work that we publish. This form provides structure for consistency and transparency in reporting. For further information on Nature Portfolio policies, see our Editorial Policies and the Editorial Policy Checklist.

Statistics

For all statistical analyses, confirm that the following items are present in the figure legend, table legend, main text, or Methods section.

n/a	Confirmed	
☐	☒	The exact sample size (*n*) for each experimental group/condition, given as a discrete number and unit of measurement
☐	☒	A statement on whether measurements were taken from distinct samples or whether the same sample was measured repeatedly
☐	☒	The statistical test(s) used AND whether they are one- or two-sided<br>*Only common tests should be described solely by name; describe more complex techniques in the Methods section.*
☐	☒	A description of all covariates tested
☐	☒	A description of any assumptions or corrections, such as tests of normality and adjustment for multiple comparisons
☐	☒	A full description of the statistical parameters including central tendency (e.g. means) or other basic estimates (e.g. regression coefficient) AND variation (e.g. standard deviation) or associated estimates of uncertainty (e.g. confidence intervals)
☐	☒	For null hypothesis testing, the test statistic (e.g. *F*, *t*, *r*) with confidence intervals, effect sizes, degrees of freedom and *P* value noted<br>*Give P values as exact values whenever suitable.*
☒	☐	For Bayesian analysis, information on the choice of priors and Markov chain Monte Carlo settings
☒	☐	For hierarchical and complex designs, identification of the appropriate level for tests and full reporting of outcomes
☐	☒	Estimates of effect sizes (e.g. Cohen's *d*, Pearson's *r*), indicating how they were calculated

Our web collection on statistics for biologists contains articles on many of the points above.

Software and code

Policy information about availability of computer code

Data collection

Flow Cytometric analysis was performed using CantoII and Fortessa FACS machine, equipped with FACSDiva Software (8.0.1).
Cell counting was performed using Cedex XS Analyzer (Roche) and CASY Cell Counter and Analyzer (OMNI Life Sciences).
Cell proliferation was analyzed using Incucyte Live-Cell Analysis System, equipped with IncuCyte ZOOM Software (2018B, Sartorius) and Cytation C10 Confocal Imaging Reader (Agilent).
qPCR was performed usinga Real-Time PCR Detection System Cycler (Applied Biosciences)
Ketosis was assessed using a handheld ketometer (Go-Keto).
16s sequencing: DNA quantity and quality were assessed using a NanoDrop Microvolume Spectrophotometer (ThermoFischer Scientific).
Sequencing of the V3 and V4 regions of prokaryotic 16S rRNA gene was performed on the Illumina MiSeq platform using 2x300 bp paired-end reads.
WGS: DNA library quantity and quality were assessed with the Agilent 2100 BioAnalyzer. Libraries were sequenced as 2x150 base pair read lengths on an Illumina NextSeq500 or NextSeq2000 platform.

LC-MS/GC-MS:
Hydrophilic interaction chromatography (HILIC) was done on a Thermo Q Exactive HF mass spectrometer (ThermoFisher Scientific) equipped with SeQuant ZIC-pHILIC 20x2.1mm columns (Merck Millipore).
For untargeted GC-MS of murine plasma samples, metabolite derivatization was performed using a multi-purpose sampler (Gerstel) and data were acquired on 7890B GC (Agilent Technologies) coupled to a 5977A mass selective detector (MSD, Agilent Technologies) or Vanquish UHPLC (ThermoFisher Scientific), coupled to a Q Exactive HF mass spectrometer (ThermoFisher Scientific).
Targeted and absolute quantitative SCFA detection in mouse fecal samples were analyzed on an 8890 GC (Agilent Technologies), coupled to an 5977B MSD (Agilent Technologies). Mass spectrometric data was acquired with MassHunter GC/MS Data Acquisition software (Version

10.0, Build 10.0.384.1).
Bile acids were analysed using UHPLC-MS/MS, consisting of an ExionLC (Sciex) coupled to a QTrap 5500 mass spectrometer (Sciex).
Long chain fatty acid extracts were analyzed on an Agilent 1290 LC coupled to an Agilent 6560 Q-TOF MS system equipped with a Dual Agilent Jet Stream ESI source using Agilent Mass Hunter LC/MS Data Acquisition (version B.09.00, Build 9.0.9044.0).

Data analysis

Image J 1.53K was used for colonic tumor surface area measurements.
FlowJo software 10.6.1 was used for flow cytometry data analysis.
Raw 16S rRNA gene sequences (FASTAQ files) were cleaned and clustered as amplicon sequence variant (ASV) count table using the Dada2pipeline and using SILVA train set (versions 132 to 138) for taxonomy inference. Differential analysis and graphical representation were performed in R, using the packages from Bioconductor (DESeq2, phyloseq) and tidyverse (ggplot).
WGS (human): Raw genome sequences (FASTQ files) were cleaned using the Sunbeam pipeline. Function profiling was performed with HUMAnN Version 3.0 using the quality-controlled and host-decontaminated FASTQ files obtained from Sunbeam. Graphical representation was performed in R, using the packages from tidyverse, broom and RColorBrewer.
WGS (mouse): The Integrated Meta-omic Pipeline (IMP; v3 - commitID #6f1badf7) was used to process paired forward and reverse reads using the built-in metagenomic workflow. The workflow includes preprocessing, assembly, genome reconstruction and functional annotation of genes based on custom databases in a reproducible manner. De novo assembly was performed using the MEGAHIT (version 2.0) assembler. Default IMP parameters were retained for all samples. MetaBAT2 and MaxBin2 in addition to binny were used for binning and genome reconstructions, i.e. MAGs. Subsequently, a non-redundant set of MAGs was obtained using DASTool v1.1.461 with a score threshold of 0.7 for downstream analyses, and those with a minimum completion of 90% and less than 5% contamination as assessed by CheckM v1.1.3. Taxonomy was assigned to the MAGs using the extensive database packaged with gtdbtk v2.0.1. In addition, Kraken2 implemented in IMP was used for taxonomic assignment of reads. For the analyses of functional potential from the assembled contigs, open-reading frames were predicted from contigs using a modified version of Prokka that includes Prodigal gene predictions for complete and incomplete open-reading frames. The identified genes were annotated with a hidden Markov models (HMM) approach, trained using an in-house database including KOs derived from the Kyoto Encyclopedia of Genes and Genomes (KEGG) database. Differential analysis was performed using the package DESeq2and using Wilcoxon rank sum test performed on CLR-transformed Kraken2 reads counts. Non-metric dimensional scaling (NMDS) analyses were performed on robust Aitchison distances calculated from Kraken2 read counts using the R package vegan (version 2.6-6.1). For further analysis as well as graphical representation R packages ggplot2, ggpubr (version 0.6.0), tidyverse, gghx4 and RColorBrewer (version 1.1-3) were used. Details on the workflows and scripts can be found on https://gitlab.com/uniluxembourg/fstm/dlsm/mdm/tsenkova_et_al_2024.

LC-MS: Raw data files obtained through LC-MS of human stool and murine plasma and stool samples were processed in TraceFinder (version 5.1.203.0) for peak identification and annotation. Three different in-house libraries, generated with reference standards, as well as one commercially available library, zCloud Offline for mzVault 2.3_Omics_2020A.db, in the Advanced Mass Spectral Database (AMSD) (HighChem, from ThermoFischer Scientific), were used for peak annotation, with the adduct formulas [M+H]+ and [M-H]-. Annotated features and integration tables were exported for post-processing.
GC-MS: Raw data files obtained through GC-MS murine plasma and stool samples were processed in MetaboliteDetector (version 3. 220190704) for peak identification and annotation. An in-house library, generated with reference standards was used for peak annotation. Deconvolution settings were applied as follows: peak threshold = 5, minimum peak height = 5, bins per scan = 10, deconvolution width = 5 scans, no baseline adjustment, minimum 15 peaks per spectrum and no minimum required base peak intensity. The data was normalized using the response ratio of the integrated peak area of each metabolite and the integrated peak area of the IS, as described in 75. Annotated features and integration tables were exported for post-processing.
For murine plasma metabolites identified in the SPF experiment, data was manually curated based on the pooled sample, spiked with the ISM. Features, which could not be confirmed through MS2 data (from the commercially available database) or through the ISM were filtered out. Metabolites with missing (N/A) values were also filtered out. Single metabolite intensities were normalized to the total metabolite area and corrected using the mean value of total metabolite values from all samples. LC-MS and GC-MS data were unified, and duplicate metabolites were filtered out, based on reliability and consistency of the peak areas. Human stool sample data was similarly processed (but without the element of data unification between methods). For murine plasma metabolites identified in the GF experiment (GC-MS), metabolites with missing (N/A) values were filtered out.
Murine stool SCFA content was quantified based on an IS and using the MassHunter Quantitative Analysis Software (version 10.2.733.8). In the LC-MS and GC-MS datasets, metabolites with over 20% missing (N/A) values were filtered out. Data from murine stool untargeted LC-MS, GC-MS and SCFA quantification was unified and duplicate metabolites were filtered out, based on reliability and consistency of the peak areas.
Raw data obtained through fatty acid LC-MS profiling were processed in Agilent Mass Hunter Profinder (ver 10.0 SP1, Build 10.0.10142.1). Target compounds were identified by exact mass (mass error ±5 ppm), isotopic pattern and retention time (±0.15 min) matching (Batch Targeted Feature Extraction). Semi-quantification was based on integrated peak area of the deprotonated target compound and normalization to internal standards.
Univariate statistical analysis of metabolomics data with Benjamini-Hochberg false discovery rate (BH FDR, performed for all datasets), principal component analysis (PCA), scaling, centering and graphical representation in the form of a heatmap (ComplexHeatmap R package) or log2foldchanges were performed in R version 4.2.1 and Rstudio version 1.4.1717.

RT-qPCR data was analyzed using qBase+ 3.2 (Biogazelle) according to MIQE guidelines.
GraphPad Software version 9.4.1 was used for all other statistical analysis and graphical representation.
All scripts and codes used are available on https://gitlab.com/uniluxembourg/fstm/dlsm/mdm/tsenkova_et_al_2024.

For manuscripts utilizing custom algorithms or software that are central to the research but not yet described in published literature, software must be made available to editors and reviewers. We strongly encourage code deposition in a community repository (e.g. GitHub). See the Nature Portfolio guidelines for submitting code & software for further information.

Data

Policy information about availability of data

All manuscripts must include a data availability statement. This statement should provide the following information, where applicable:

- Accession codes, unique identifiers, or web links for publicly available datasets
- A description of any restrictions on data availability
- For clinical datasets or third party data, please ensure that the statement adheres to our policy

The below data availability statement has been included in the methods section of this manuscript.
Raw sequencing data files for 16S gene sequencing from murine fecal samples are available at the ENA's sequence archive under the accession PRJEB70917 [https://www.ebi.ac.uk/ena/browser/view/PRJEB70917] (GF) and PRJEB70920 [https://www.ebi.ac.uk/ena/browser/view/PRJEB70920] (SPF). Raw sequencing data files for 16S gene sequencing from human stool samples are available at the ENA's sequence archive under the accession PRJEB70932 [https://www.ebi.ac.uk/ena/browser/view/PRJEB70932]. Raw sequencing data samples for mouse stool samples are available at the ENA's sequence archive under the accession PRJEB66281 [https://www.ebi.ac.uk/ena/browser/view/PRJEB66281]. Raw sequencing data files for RNAseq from LCFA-treated HT29 cells can be accessed under ArrayExpress accession number E-MTAB-14429 [https://www.ebi.ac.uk/biostudies/arrayexpress/studies/E-MTAB-14429]. Raw sequencing data files for WGS from human stool samples have been filtered for human reads and filtered data are available at the ENA's sequence archive under the accession number PRJEB84188.
Metabolomics data processed with Tracefinder are available at the NIH Common Fund's National Metabolomics Data Repository (NMDR) website, the Metabolomics Workbench, https://www.metabolomicsworkbench.org. Untargeted LC-MS profiling data of donor stool samples have been assigned Study ID ST003681, untargeted LC-MS profiling data of fecal samples from SPF diet experiment have been assigned Study ID ST003679, untargeted LC-MS profiling data of plasma samples from SPF diet experiment have been assigned Study ID ST003683 and untargeted LC-MS profiling of fecal samples from CMT experiment have been assigned Study ID ST003680. The data can be accessed directly via its Project DOI: http://dx.doi.org/10.21228/M8HZ6G.
Source data are provided with this paper.

The code is available on: https://gitlab.com/uniluxembourg/fstm/dlsm/mdm/tsenkova_et_al_2024

Research involving human participants, their data, or biological material

Policy information about studies with human participants or human data. See also policy information about sex, gender (identity/presentation), and sexual orientation and race, ethnicity and racism.

Reporting on sex and gender	Sex of fecal sample donor has been reported in Extended Data Figure 1, but no sex-based analyses have been performed. Gender has not been reported.
Reporting on race, ethnicity, or other socially relevant groupings	Race, ethnicity and other socially relevant groupings have not been reported.
Population characteristics	The ages of stool sample donors have been reported in Extended Data Figure 1
Recruitment	All healthy donors from the MuSt study were enrolled at the Centre Hospitalier de Luxembourg (Luxembourg, Luxembourg).
Ethics oversight	Samples were donated willingly under informed consent and were handled in accordance with institutional guidelines. Ethical approval was given by the Comité National d'Ethique de Recherche of Luxembourg (MuSt authorization number 201110/05) and the National Commission for Data Protection in Luxembourg.

Note that full information on the approval of the study protocol must also be provided in the manuscript.

Field-specific reporting

Please select the one below that is the best fit for your research. If you are not sure, read the appropriate sections before making your selection.

☒ Life sciences ☐ Behavioural & social sciences ☐ Ecological, evolutionary & environmental sciences

For a reference copy of the document with all sections, see nature.com/documents/nr-reporting-summary-flat.pdf

Life sciences study design

All studies must disclose on these points even when the disclosure is negative.

Sample size	For in vitro studies a minimum of triplicates per condition were used and each experiment was verified in at least three independent experiments. For in vivo work, the number of mice per group was calculated based on a power calculation in liaison with a bio-statistician. Please refer to the Figure legends and Method sections for further details.
Data exclusions	No data was excluded unless clear technical issues were identified.
Replication	All in vitro experiments were verified in at least three independent experiments with same overall outcome. Specific number of repetitions is provided in the figure legends of the manuscript. For in vivo groups, adequate group size was calculated beforehand. As stated in the manuscript, for some experiments, we pooled two independent experiments to reach the number calculated by the bioinformatician.

Randomization	Before starting all of the in vivo experiments, mice were randomly allocated to different groups. All Mass Spec samples and DNA sequencing samples were randomized during the analysis to avoid bias from ex. instrument drift.
Blinding	Analyses of in vivo samples (tumor counts) were blinded and analyzed by two researchers independently. IDs were uncovered after final result was obtained. Results from both persons lead to the same overall result.

Reporting for specific materials, systems and methods

We require information from authors about some types of materials, experimental systems and methods used in many studies. Here, indicate whether each material, system or method listed is relevant to your study. If you are not sure if a list item applies to your research, read the appropriate section before selecting a response.

Materials & experimental systems

n/a	Involved in the study	
☐	☒	Antibodies
☐	☒	Eukaryotic cell lines
☒	☐	Palaeontology and archaeology
☐	☒	Animals and other organisms
☒	☐	Clinical data
☒	☐	Dual use research of concern
☒	☐	Plants

Methods

n/a	Involved in the study	
☒	☐	ChIP-seq
☐	☒	Flow cytometry
☒	☐	MRI-based neuroimaging

Antibodies

Antibodies used

Antibody, Isotype, Reactivity, Conjugate, Clone and Supplier are listed below.
CD11b Rat IgG2β Mouse APC-Cy7 M1/70 BioLegend
CD11b Rat IgG2β Mouse PB M1/70 BioLegend
CD11c Hamster IgG1 Mouse PE-Cy7 HL3 BD Biosciences
CD127 Rat IgG2β Mouse PE-Cy7 SB/199 BD Biosciences
CD16/32 Rat IgG2β Mouse Purified 2.4G2 BD Biosciences
CD19 Rat IgG2α Mouse PE 1D3 BD Biosciences
CD19 Mouse IgA Mouse APC-Cy7 MB19-1 ThermoFisher Scientific
CD25 Rat IgG1 Mouse PE PC61 BD Biosciences
CD28 Hamster Mouse Purified 37.51 BioLegend
CD3 Rat IgG2β,κ Mouse APC-eFluor780 17A2 eBioscience (Invitrogen)
CD3 Hamster IgG Mouse Purified 145-2C11 BioLegend
CD3e Hamster IgG1 Mouse FITC 145-2C11 BD Biosciences
CD4 Rat IgG2α Mouse BV786 RM4-5 BD Biosciences
CD4 Rat IgG2α Mouse FITC RM4-5 BD Biosciences
CD40 Rat IgG2α Mouse PE 3/23 BD Biosciences
CD45.2 Mouse IgG2α Mouse FITC 104 eBioscience (Invitrogen)
CD5 Rat IgG2α Mouse APC-eFluor780 53-7.3 ThermoFisher Scientific
CD8 Rat IgG2β,κ Mouse PB 53-6.7 BD Biosciences
CD8 Rat IgG2β,κ Mouse APC-eFluor780 53-6.7 eBioscience (Invitrogen)
CD80 Hamster IgG2 Mouse FITC 16-10A1 BD Biosciences
CD86 Rat IgG2α Mouse BV650 GL1 BD Biosciences
CD8a Rat IgG2α Mouse BUV395 53-6.7 BD Biosciences
CD90.2 Rat IgG2α,κ Mouse SB600 53-2.1 eBioscience (Invitrogen)
EOMES Rat IgG2α,κ Mouse PE-Cy7 DAN11MAG eBioscience (Invitrogen)
F4/80 Rat IgG2α Mouse PE-Cy7 BM8 ThermoFisher Scientific
FcεR1 Hamster IgG Mouse APC-eFluor780 MAR-1 ThermoFisher Scientific
FoxP3 Rat IgG2α Mouse APC FJK-16s ThermoFisher Scientific
GATA3 Rat IgG2β Mouse PE 16E10A23 BioLegend
I-A/I-E Rat IgG2β Mouse APC M5/114.15.2 BioLegend
IFNγ Rat IgG1 Mouse APC XMG1.2 BD Biosciences
IFNγ Rat IgG1, κ Mouse Purified XMG1.2 BD Biosciences
IL-17a Rat IgG1 Mouse BV605 TC11-18H10 BD Biosciences
IL-2 Rat IgG2β Mouse PB JES6-5H4 BD Biosciences
IL-22 Goat IgG Mouse PE Polyclonal BioLegend
IL-4 Rat IgG1 Mouse PE-Cy7 11B11 BioLegend
Ly6G Rat IgG2β Mouse APC-Cy7 RB6-8C5 ThermoFisher Scientific
NK1.1 Mouse IgG2α, κ Mouse eFluor450 PK136 eBioscience (Invitrogen)
NKp46 Rat IgG2α Mouse BUV737 29A1.4 BD Biosciences
RORγT Rat IgG1 Mouse APC B2D ThermoFisher Scientific

T-bet Rat IgG2β Mouse PB 4B10 BioLegend
TCRβ Hamster IgG Mouse APC-eFluor780 H57-597 eBioscience (Invitrogen)
TER119 Rat IgG2β,κ Mouse APC-Cy7 TER-119 BioLegend
TNFα Rat IgG1 Mouse BV510 MP6-XT22 BD Biosciences
Occludin Rabbit IgG Mouse Purified E6B4R Cell Signalling

Validation	All used antibodies are well described and used across research laboratories as well as used in published manuscripts. Further information can be found on the corresponding manufacturer websites.

Eukaryotic cell lines

Policy information about cell lines and Sex and Gender in Research

Cell line source(s)	HCT116 (CCL-247), HT-29 (HTB-38) and Caco-2 (HTB-37) CRC cell lines were obtained from ATCC and maintained in DMEM-F12 with 10% [v/v] foetal bovine serum (FBS) and 1% [v/v] penicillin/streptomycin.
Authentication	Cell lines were and authenticated before their use in this study (STR analysis, DSMZ).
Mycoplasma contamination	Cell lines were mycoplasma-free .
Commonly misidentified lines (See ICLAC register)	HCT116 are part of this list, but have been authenticated via STR profiling at DSMZ before the study.

Animals and other research organisms

Policy information about studies involving animals; ARRIVE guidelines recommended for reporting animal research, and Sex and Gender in Research

Laboratory animals	Mus musculus C57BL/6J and C57BL6/NTac mice aged between 9 and 13 weeks were used in this study.
Wild animals	No wild animals were used.
Reporting on sex	Male and female mice were used for all experiments.
Field-collected samples	No field collected samples were used.
Ethics oversight	Animal experiments were performed according to all applicable laws and regulations, with approval from the Animal Experimentation Ethics Committee (AEEC) and the veterinary service of the Ministry of Agriculture, Viticulture and Rural Development of Luxembourg (LUPA2019-13). They ensure that the care and the use of the animals for research purposes is conducted according to the European Union Directive 2010/63/EU and to the Grand-Ducal Regulation of the 11th of January 2013, regarding the protection of animals used for experimentation. This includes justification for the use of the animals, guidelines for their care and welfare, and the incorporation of the 3Rs (replacement, reduction and refinement). All animal protocols were reviewed by a biostatistician.

Note that full information on the approval of the study protocol must also be provided in the manuscript.

Plants

Seed stocks	n/a
Novel plant genotypes	n/a
Authentication	n/a

Flow Cytometry

Plots

Confirm that:

- [x] The axis labels state the marker and fluorochrome used (e.g. CD4-FITC).
- [x] The axis scales are clearly visible. Include numbers along axes only for bottom left plot of group (a 'group' is an analysis of identical markers).
- [x] All plots are contour plots with outliers or pseudocolor plots.
- [x] A numerical value for number of cells or percentage (with statistics) is provided.

Methodology

Sample preparation

Lamina propria immune cell isolation (for Supplementary Figure 2)
Colons (opened longitudinally) were cut into 2cm-long pieces, placed in a 50mL Falcon tube containing 20mL of strip medium (RPMI GlutaMAX® (Gibco), 3%FBS (Lonza), 1%P/S (Gibco), 5mM EDTA (Sigma-Aldrich), 0.154mg/mL DTT (Merck)) and two sterile metallic beads, and incubated in a bacterial shaker at 800rpm, 37°C for 20 minutes. After incubation, the strip medium and intestinal pieces were poured through a metallic kitchen strainer placed on top of a 250mL glass beaker. The flow through (strip medium) was collected, filtered through a 70µM cell strainer placed on top of a 50mL Falcon tube, and then centrifuged at 400g for five minutes at 4°C. The supernatant was discarded, the cell pellet was resuspended in 1mL of RPMI GlutaMAX®, 10% FBS, 1%P/S and stored on ice until seeding in a 96-well plate and staining. This fraction contains the intraepithelial lymphocytes (IEL). The intestinal pieces were transferred to a 50mL Falcon tube containing 10mL of shake medium and underwent three 30-second-long manual shaking steps, with a refreshment of the shake medium for each shake. They were then poured into the kitchen strainers and washed with cold sterile PBS to remove the EDTA. The intestinal pieces were transferred into a 50mL glass beaker in 10mL of digestion medium and cut into very small pieces. The beakers were covered with parafilm and incubated for 30 minutes at 800 rpm, 37°C in a bacterial shaker. After digestion, the beakers were placed on ice and 20 mL of cold RPMI GlutaMAX® 3% FBS 1% P/S was added to each beaker to stop the reaction. The intestinal pieces and the digestion media were pressed through a 70µM cell strainer using a plunger from a 5mL syringe, and the filter was washed with an additional 20mL of RPMI GlutaMAX® 3% FBS 1% P/S. The tubes were centrifuged at 400g for five minutes at 4°C, the supernatant was discarded, and the cell pellet was resuspended in 10mL RPMI GlutaMAX® 3% FBS 1% P/S and filtered through a 40µM cell strainer. The tubes were centrifuged at 400g for five minutes at 4°C, the supernatant was discarded, and the cell pellet was resuspended in 1mL RPMI GlutaMAX® 10% FBS 1% P/S and stored on ice until seeding in a 96-well plate and staining.

Lamina propria immune cell isolation (for all other experiments)
Colons (opened longitudinally) were cut into one-cm-long pieces and processed according to the Mouse Lamina Propria Dissociation Kit (Miltenyii) instructions, with the following modifications: 1) a bacterial shaker was used (800rpm, 37°C) instead of a MACSmix tube rotator. 2) 70µM cell strainers were used instead of 100µm cell strainers. After obtaining the final cell pellet, cells were resuspended in 1.5mL of RPMI GlutaMAX® 10% FBS 1%P/S per spleen and stored on ice until seeding in 96-well plates.

Spleen immune cell isolation
Spleens were resected at endpoint and transported to the laboratory in RPMI GlutaMAX® 10% FBS on ice. Each spleen was injected with 1mL of RPMI GlutaMAX® 1%P/S, 5mg/mL Liberase (Merck Life Sciences), 25mg/mL DNase (Sigma-Aldrich), cut up into small pieces (<1mm2) and incubated at 37°C for 30 minutes. After incubation, spleens were pressed though a 70µM cell strainer using a plunger from a 5mL syringe, and the filter was washed with 20mL of RPMI GlutaMAX® 10% FBS 1%P/S. The cell suspension was centrifuged at 400g for five minutes at 4°C, the supernatant was discarded, and the cell pellet was resuspended in 3mL RPMI GlutaMAX® 10% FBS 1%P/S per spleen and stored on ice until seeding in 96-well plates.

Mesenteric lymph node immune cell isolation
MLNs were pressed through a 70µM cell strainer using a plunger from a 5mL syringe, and the filter was washed with 20mL of RPMI GlutaMAX® 10% FBS 1% P/S. The cell suspension was centrifuged at 400g for 5 minutes at 4°C, the supernatant was discarded, and the cell pellet was resuspended in 1 mL of RPMI GlutaMAX® 10% FBS 1%P/S per spleen and stored on ice until seeding in 96-well plates.

Immune cell restimulation
100µL of the cell suspensions obtained from the digestion of the colons (lamina propria and IELs), the spleens and the MLNs were distributed per well in a 96-well plate. Cells which did not require restimulation were immediately processed as described below. 100µL of 100ng/mL PMA (Sigma-Aldrich) 3µg/mL ionomycin (Alfa Aesar), RPMI GlutaMAX® 10% FBS 1% P/S was added per well to the plates requiring restimulation (those panels which include intracellular cytokine stains). These plates were incubated for one hour at 37°C, then Brefeldin A (dilution of 1:1000, 1x final concentration, eBioscience (Invitrogen)) was added to each well, then the incubation continued for another three hours. After a total restimulation time of four hours, the cells were further processed as described below.

Immune cell staining and acquisition (FACS)
The plates were centrifuged, the supernatants were discarded, and the cells were resuspended in 50µL/well extracellular antibody mixes (LIVE/DEAD Fixable Near-Infrared Dead Cell Stain Kit (ThermoFisher Scientific) 1:1000, antibodies 1:100 in FACS buffer) and incubated for 30 minutes at 4°C or overnight at 4°C. For the staining of macrophages and dendritic cells (DC), cells were resuspended in 25µL/well of CD16/32 (1:50, L/D 1:1000 in FACS buffer) and incubated for 15 minutes at 4°C, before the addition of 25µL/well of extracellular antibody mixes (antibodies 1:50, L/D 1:1000 in FACS buffer). After

incubation, cells were washed with 200µL/well FACS buffer (centrifugation for five minutes at 400g, 4°C) and the supernatant was discarded. Cells were then fixed and permeabilized using the Cytofix/Cytoperm Kit (BD Biosciences), according to the kit manual. After permeabilization, cells were resuspended in 50µL/well intracellular antibody mixes (antibodies 1:100 in Perm Buffer (from Cytofix/Cytoperm Kit)) and incubated for 30 minutes at 4°C. After incubation, cells were washed with Perm Buffer, and resuspended in 100µL Perm Buffer for FACS acquisition.

T cell differentiation assay

Spleens and lymph nodes from young (<8-week-old) male and female C57BL/6J mice were isolated using the CD4+ T Cell Isolation Kit (Miltenyii Biotec) and transported to the laboratory in PBS on ice. The organs were pressed through 70µM cell strainers using plungers from 5mL syringes and washed with 10mL of MACS buffer (PBS 1x, 1mM EDTA, 1% FBS (sterile), centrifugation at 300g for 10 minutes). Cells were resuspended in 200µL of MACS buffer per sample and 50µL of CD4 T cell biotin antibody mix was added to each sample. After six minutes of incubation at 4°C, 100µL of MACS buffer was added to each sample and 50µL of Anti-Biotin Microbeads and 100µL of CD44 Microbeads were added to each sample. After 12 minutes of incubation at 4°C, the samples were washed with 5mL of MACS buffer. The cell pellets were resuspended in 500µL of MACS buffer and run through LS columns (negative sorting, Miltenyii Biotec). Cells were spun down, supernatant was discarded and the cell pellets were resuspended in 1mL of T cell media. Cells were counted using a Neubauer improved counting chamber (VWR). 50µL of a 4x concentrated Th17 cell differentiation cytokine mix (final concentrations: 2ng/mL TGF-β (R&D systems), 30ng/mL IL-6 (Miltenyii Biotec), 5µg/mL anti-IFNγ and 1µg/mL anti-CD28) were added per well in a 96-well plate pre-coated with 100µL of anti-CD3 5ug/mL per well (incubated for at least three hours at 37°C). 50µL of 4x concentrated treatments of interest (final concentrations: 1%, 2% and 5% lipid mixture (Sigma-Aldrich), 10µm BSA (Carl ROTH), 10µm BSA-conjugated palmitic (Agilent Technologies) and oleic acid (Sigma-Aldrich), 10mM, 50mM and 100mM β-hydroxybutyrate sodium salt (β-HB, Merck), 1mM, 10mM and 100mM acetone (Sigma-Aldrich), 10mM, 100mM and 250mM NaAcAc (homemade)) were added per well. 200 000 cells in 100µL were seeded per well, on top of the cytokine mix and treatments. Three days later, cells were centrifuged at 300g for five minutes, the supernatant was removed, and the cells were resuspended in 200µL of 50ng/mL PMA 1.5µg/mL ionomycin in T cell media per well. They were incubated for one hour at 37°C, then Brefeldin A (dilution 1:1000) was added to each well, then the incubation continued for another three hours. After a total restimulation time of four hours, the cells were stained as described above.

Instrument

BD FACS CantoII and BD LSR Fortessa FACS machines were used for sample acquisition.

Software

BD FACS Diva 8.0.1 and FlowJo 10.6.1 softwares were used for data analysis.

Cell population abundance

The cellular concentration of all cell suspensions was quantified using the Cedex XS Analyzer (Roche), by mixing 10µL of cells with 10µL of Trypan Blue solution (Roche) and loading 10µL into the Cedex Smart Slide (ibidi) channel; or using the CASY Cell Counter and Analyzer (OMNI Life Sciences), by diluting 10µL of cell suspension in 10mL of CASYton (OMNI Life Sciences) solution.

Gating strategy

FSC/SSC selection of cells, exclusion of doublets by FSC-A/FSC-H, selection of live cells based on Live/Dead staining.
Unstained controls and single stained controls were acquired to identify negative and positive cell populations for each marker.
Detailed gating strategies are provided in extended data figures 9 and 10.

☒ Tick this box to confirm that a figure exemplifying the gating strategy is provided in the Supplementary Information.
